# Gut microbiota-involved mechanisms in enhancing systemic exposure of ginsenosides by coexisting polysaccharides in ginseng decoction

**DOI:** 10.1038/srep22474

**Published:** 2016-03-02

**Authors:** Shan-Shan Zhou, Jun Xu, He Zhu, Jie Wu, Jin-Di Xu, Ru Yan, Xiu-Yang Li, Huan-Huan Liu, Su-Min Duan, Zhuo Wang, Hu-Biao Chen, Hong Shen, Song-Lin Li

**Affiliations:** 1 Department of Pharmaceutical Analysis, Hospital of Integrated Traditional Chinese and Western Medicine Affiliated to Nanjing University of Chinese Medicine, Nanjing, Jiangsu, PR China; 2 Department of Metabolomics, Jiangsu Province Academy of Traditional Chinese Medicine and Jiangsu Branch of China Academy of Chinese Medical Sciences, Nanjing, Jiangsu, PR China; 3 School of Chinese Medicine, Hong Kong Baptist University, Hong Kong; 4 State Key Laboratory of Quality Research in Chinese Medicine, Institute of Chinese Medical Sciences, University of Macau, Macao

## Abstract

Oral decoctions of traditional Chinese medicines (TCMs) serve for therapeutic and prophylactic management of diseases for centuries. Small molecules and polysaccharides are the dominant chemicals co-occurred in the TCM decoction. Small molecules are well-studied by multidisciplinary elaborations, whereas the role of polysaccharides remains largely elusive. Here we explore a gut microbiota-involved mechanism by which TCM polysaccharides restore the homeostasis of gut microbiota and consequently promote the systemic exposure of concomitant small molecules in the decoction. As a case study, ginseng polysaccharides and ginsenosides in *Du-Shen-Tang*, the decoction of ginseng, were investigated on an over-fatigue and acute cold stress model. The results indicated that ginseng polysaccharides improved intestinal metabolism and absorption of certain ginsenosides, meanwhile reinstated the perturbed holistic gut microbiota, and particularly enhanced the growth of *Lactobacillus* spp. and *Bacteroides* spp., two major metabolic bacteria of ginsenosides. By exploring the synergistic actions of polysaccharides with small molecules, these findings shed new light on scientization and rationalization of the classic TCM decoctions in human health care.

Traditional Chinese medicines (TCMs) are used for disease prevention and treatment through the ages, and are thought to have profound impacts on human survival and reproduction^1^,^2^. During long-term empirically clinical use, TCMs are mostly prepared by boiling with water to generate decoction (water extracts) for oral administration. However, scientific connotations and mysteries of TCM decoction are still largely veiled. For example, completely differing from Western medicines, chemical compositions of which are simplex and definite, TCM decoction normally has several kinds of chemical components. Which components contribute to therapeutic effects of the decoction and how they synergistically work remain unknown. Illumination of these issues would be significant for the inheritance and innovation of TCM decoctions^3^.

Chemical diversity of TCM decoctions has been well-defined by accumulated phytochemical studies, small molecules (generally MW < 1 kDa) and polysaccharides are among the most representative^4^,^5^. Intensive multidisciplinary research provides abundant information of small molecules in TCMs by elucidating chemical structures, evaluating pharmacological activities, determining systemic exposure, as well as exploring action targets^6^,^7^,^8^,^9^. As a typical example, it has been adequately demonstrated that glycosides, such as saponins, iridoid glycosides and flavone glycosides, which are normally polar chemicals and occur frequently in TCM decoctions^10^,^11^,^12^, are often metabolized to secondary glycosides and/or aglycones with better bioavailability and bioactivity by enzymes encoded in intestinal bacteria (frequently probiotics)^13^,^14^,^15^,^16^. By sufficient elaborations, small molecules are commonly deemed to be the crucial bioactive chemicals that are responsible for curative effects of TCM decoction. By contrast, the role of TCM polysaccharides is not clear yet as they are generally indigestible by oral administration and hardly absorbable in the gastrointestinal tract^17^. Due to the confined scientific perception, nowadays TCM polysaccharides are always under-appreciated or even disregarded. For example, in modern industrialized TCM preparation, polysaccharides are normally removed as impurities to meet the requirements on purity and dosage amounts of the final products^18^; likewise, scientific research on TCM decoctions also excluded polysaccharides from biologically key chemicals^19^,^20^,^21^. Obviously, suchlike situations not only deviate from the traditional usage of TCM, but also lack scientific evidences. If and how polysaccharides in TCM decoctions therapeutically contribute after oral administration warrant further investigation.

Multiple indigestible dietary carbohydrates, such as oligofructose, galactooligosaccharides, lactulose and inulin (long chain β-fructan), are proven as prebiotics to selectively stimulate the growth of a subset of beneficial gut bacteria (probiotics), and consequently to sustain the homeostasis of gut microbial community as well as the host health^17^,^22^. However, to our knowledge, such attentions have not been paid to TCM polysaccharides thus far. Besides, it has been fully evidenced that various diseases, such as obesity, diabetes and cancer, are able to change the compositions of gut microbiota^23^, and that both the pathological symptoms and the gut microbiota dysbiosis can be alleviated by TCMs, including TCM polysaccharides, although such functional connections are still less well understood^21^,^24^. These facts described above encouraged us to address the hypothesis that polysaccharides in TCM decoction, although indigestible when orally administered, potentially work directly (as prebiotics) and/or indirectly (under certain pathological conditions) to induce favorable changes in the intestinal microbiota. Then the improved gut microbiota further enhances intestinal metabolism and absorption of the bioactive small molecular chemicals co-administered in the TCM decoction. Here we aim to experimentally test the extrapolated gut microbiota-involved synergistic actions between polysaccharides and small molecules coexisted in TCM decoction.

*Du-Shen-Tang* (DST), the decoction of ginseng that was first documented in “*Shi Yao Shen Shu*” (Book on Ten Magic Herbs) in the year 1348 of Yuan Dynasty, is a well-known TCM prescription for medical emergency of *Qi* (vital energy)-deficiency with over 600-year history of clinical application^25^. In modern clinical practice, DST is prescribed for treatments of cardiogenic shock and dilated cardiomyopathy, etc^26^. Chemical composition of DST is dominated by ginsenosides and polysaccharides, in which ginsenosides, as a common type of glycosides, have been intensively demonstrated to possess multiple cardiovascular activities, for example, reducing platelet adhesion, vasomotor regulation, improving lipid profiles and influencing various ion channels^27^. More interestingly, better bioavailability and bioactivity of some secondary ginsenosides generated by intestinal microbiota metabolism compared with those of their primary ginsenosides are reported^28^. Therefore, ginsenosides are generally recognized as the crucial bioactive components of ginseng as well as DST^29^. However, ginseng polysaccharides are less studied, and their role in DST needs to be further defined^30^. In this study, DST was selected as an example to verify the hypothesis proposed above (Fig. 1). A rat model of *Qi*-deficiency with abnormality (dysbiosis) in the composition of gut microbiota was established by suffering successive over-fatigue and acute cold stress (OACS). On the model rats, we examined effects of ginseng polysaccharides on the intestinal metabolism and absorption of ginsenosides, and then explored the gut microbiota-mediated mechanisms.

## Results

### Chemical characterization of ginseng polysaccharides and ginsenosides in DST

The prepared ginseng polysaccharides were chemically characterized in terms of molecular weight distribution and monosaccharide compositions. The results were summarized in Supplementary Figure 1. The high-performance gel permeation chromatography (HPGPC) chromatogram showed that ginseng polysaccharides had a wide molecular weight distribution from 1.00 kDa to 1308.98 kDa with the weight-average molecular weight and number-average molecular weight of 39.56 kDa and 3.57 kDa, respectively (calculated by the previously established molecular weight-retention time calibration curve^31^) (Supplementary Figure 1a). Monosaccharide composition analysis indicated that ginseng polysaccharides mainly consisted of seven monosaccharides and uronic acids, i.e. mannose (Man), rhamnose (Rha), galacturonic acid (GalA), glucose (Glc), galactose (Gal), arabinose (Ara) and fucose (Fuc) with a mole ratio of 1.11:1.00:7.11:15.98:4.40:6.12:2.42 (Supplementary Figure 1b,c).

Ginsenosides in DST were analyzed by ultra-performance liquid chromatography quadrupole time of flight mass spectrometry (UPLC-QTOF-MS). Among all identified ginsenosides (Supplementary Figure 2), eleven main chemicals, namely ginsenosides Re, Rg_1_, Rf, Rb_1_, 20(*S*)-Rg_2_, Rc, Rb_2_, Rd, F_2_, 20(*S*)-Rg_3_, CK (Supplementary Table 1), were selected as the investigated ginsenosides in this study.

### Ginseng polysaccharides reinstated OACS-induced disorder of endogenous metabolism

Effects of OACS and the associated ginseng polysaccharides intervention on endogenous metabolism of the rats were evaluated by UPLC-QTOF-MS-based metabonomics approach. Oligofructose, a well-defined prebiotic, was selected as a positive control in the entire experimental study. Both plasma and urine samples from the blank, model, ginseng polysaccharides and oligofructose groups were analyzed by UPLC-QTOF-MS, and the obtained data were then subjected to supervised orthogonal partial least squares discriminant analysis (OPLS-DA). The score plots of either plasma or urine exhibited an apparent separation between the blank and model groups, indicating that endogenous metabolism of the rats in the model group were conspicuously disturbed by OACS (Supplementary Figure 3a and 4a). The loading plot and S-plot analysis explored eight plasmatic and six urinary endogenous metabolites that were significantly altered (increased or decreased) (*p* < 0.05 or *p* < 0.01) (the plots marked by red boxes in Supplementary Figure 3b,c and 4b,c) in the model group as biomarkers for the disorder of endogenous metabolism by OACS. They were tentatively identified as tryptophan, phenylalanine, butyrylcarnitine, lysophosphatidylcholine (LPC) C16:0, LPC C18:1, LPC C18:0, cholic acid (CA), cresol sulfate (plasmatic), trimethylamine-N-oxide (TMAO), 4-hydroxyphenylacetate (4-HPPA), hippurate, citrate, isocitrate and 4-methy-phenol (4-MP) (urinary), respectively, by precise molecular mass and fragment ions according to the reported literatures (Supplementary Table 2). By treatment with ginseng polysaccharides or oligofructose, the plasmatic and urinary biomarkers were significantly (*p* < 0.05 or *p* < 0.01) reinstated in the treated groups, leaning close to normal levels measured in the blank group (Supplementary Figure 5). It suggested that ginseng polysaccharides were capable to reinstate the OACS-induced disorder of endogenous metabolism.

### Ginseng polysaccharides restored OACS-induced dysbiosis of gut microbiota

To evaluate whether OACS induces gut microbiota alterations, we surveyed the fecal bacterial population by 16S rRNA gene sequencing of samples isolated from the rats of blank and model groups. The stable species accumulation curves (Supplementary Figure 6a) and Shannon-Wiener curves (Supplementary Figure 6b) suggested that the data covered most diversity and were sufficient to meet the requirements on data analysis. Taxonomy-based analysis exhibited that gut microbiota of rats in the blank group was quantitatively dominated by bacterial phyla Bacteroidetes, Firmicutes and Proteobacteria (Fig. 2a) with relative abundances of 55.23%, 35.84% and 7.47%, respectively. However, OACS conspicuously changed the microbial composition in the model group with more Bacteroidetes (80.61%) but fewer Firmicutes (16.24%) and Proteobacteria (2.20%) (Fig. 2a). The effect of OACS on altering the gut microbiota was further evident by compositional analysis at bacterial genus level (Fig. 2b). Operational taxonomic units (OTUs) abundance based unweighted UniFrac analysis by principal coordinate analysis (PCoA) was subsequently performed to provide an overview of the variation of gut microbiota (Fig. 2d). The model cluster was distinctly away from the blank one, indicating robust differences in the membership of gut bacteria between the blank and model groups. Additionally, two crucial beneficial symbiotic bacteria, *Bacteroides* spp. and *Lactobacillus* spp., were particularly concerned, and it was found both of them were substantially decreased in the model group by OACS although not reach a significant level (Fig. 2c).

Effects of ginseng polysaccharides on the gut microbiota of OACS model were then investigated. Both ginseng polysaccharides and oligofructose reversed the OACS-induced gut microbial dysbiosis at phyla level to approach the homeostasis of blank group mainly by increasing the relative abundance of Firmicutes and decreasing that of Bacteroidetes (Fig. 2a,b). The PCoA results of OTUs further confirmed such a tendency: the clusters of the ginseng polysaccharides, oligofructose and blank groups intertwined mutually, but detached with that of the model group (Fig. 2d). The relative abundances of *Bacteroides* spp. and *Lactobacillus* spp. were increased by ginseng polysaccharides or oligofructose treatments with significant (*p* < 0.05) or near-significant (0.05 < *p* < 0.1) differences compared with the model group (Fig. 2c). Altogether, it is concluded that OACS led to dysbiosis of the gut microbiota of the rats, and ginseng polysaccharides or oligofructose treatment was able to substantially restore the dysbiosis.

### Ginseng polysaccharides enhanced OACS-attenuated systemic exposure of ginsenosides

Pharmacokinetics of the eleven ginsenosides after oral administration of the ginsenosides extracts was studied by high-performance liquid chromatography triple quadrupole mass spectrometry (HPLC-TQ-MS). Nine ginsenosides were well determined, with the exception of Rf and F_2_ that were not detected at several time points (Fig. 3). Plasma concentration-time curves of the nine ginsenosides were provided in Fig. 4, and the relevant pharmacokinetic parameters were summarized in Supplementary Table 3.

The pharmacokinetic parameters of the nine ginsenosides in the blank, model, ginseng polysaccharides and oligofructose groups were compared. Here the *C*_max_ and *AUC* with statistically significant changes were described (Fig. 5). Compared with the blank group, OACS conspicuously affected the pharmacokinetic behaviors of several ginsenosides in the model group. To be specific, *AUC* of ginsenosides 20(*S*)-Rg_2_, Rd and 20(*S*)-Rg_3_ reduced significantly about 29.67%, 47.88% and 46.73% (*p* < 0.05) in the model group (from 93.36 ± 17.58 ng·min/mL to 65.66 ± 16.00 ng·min/mL, 374.62 ± 133.91 ng·min/mL to 195.27 ± 126.23 ng·min/mL, and 126.66 ± 31.23 ng·min/mL to 67.47 ± 30.60 ng·min/mL, respectively) (Fig. 5a). Besides, significant decrease of 58.43% and 52.34% (*p* < 0.05) also occurred on *C*_max_ (*C*_max_1: 5.87 ± 2.87 μg/mL to 2.44 ± 0.96 μg/mL; *C*_max_2: 5.77 ± 2.56 μg/mL to 2.75 ± 0.87 μg/mL) of ginsenoside 20(*S*)-Rg_3_ (Fig. 5b). By treatment of ginseng polysaccharides, however, *AUC* of ginsenosides 20(*S*)-Rg_2_, Rd and 20(*S*)-Rg_3_ were respectively increased by 52.85%, 121.10% and 125.76% compared with the model group (100.36 ± 23.21 ng·min/mL, 431.74 ± 143.71 ng·min/mL and 152.32 ± 27.23 ng·min/mL, respectively) (*p* < 0.05) (Fig. 5a). Meanwhile, *C*_max_ of ginsenosides Rg_1_, Rd and 20(*S*)-Rg_3_ were also significantly (*p* < 0.05) increased compared with the model group (Fig. 5b and Supplementary Table 3).

In addition to pharmacokinetic parameter determination, fecal contents of the ginsenosides were also concerned. First, chemical profiles of 24 h feces after ginsenosides administration in the four groups were qualitatively analyzed by UPLC-QTOF-MS. Totally twenty-eight ginsenosides were detected and identified (Supplementary Figure 7 and Supplementary Table 4 and Supplementary Figure 2). Then fecal contents of the eleven ginsenosides in the four groups were quantitatively determined and compared by the developed HPLC-TQ-MS method (Supplementary Table 5). As summarized in Fig. 5c and Supplementary Table 5, accumulated excretion amounts of the eleven ginsenosides in 24 h feces differed conspicuously in the four groups. OACS significantly (*p* < 0.05) increased the contents of ginsenosides Re (6093.60 ± 6348.53 ng/g to 121491.70 ± 104632.70 ng/g, 1893.76%), 20(*S*)-Rg_2_ (16302.83 ± 10154.32 ng/g to 72229.66 ± 60461.32 ng/g, 343.05%), and Rc (45.13 ± 28.75 ng/g to 72.95 ± 35.02 ng/g, 61.64%), but deceased those of ginsenosides Rd (1410.65 ± 668.07 ng/g to 645.57 ± 484.50 ng/g, 54.24%) and Rg_1_ (218534.90 ± 315766.60 ng/g to 58256.36 ± 60478.57 ng/g, 73.34%) significantly (*p* < 0.05 or *p* < 0.01) in the model group feces. Statistically significant variation was not observed for the other seven ginsenosides contents in the feces between the blank and model groups (Supplementary Table 5). Compared with the model group, however, the content of six analytes were reduced in the feces of ginseng polysaccharides group with significant (*p* < 0.05 or *p* < 0.01) differences. With the exception of ginsenosides Re, 20(*S*)-Rg_2_, Rc, Rd that significantly changed by OACS, the contents of ginsenosides 20(*S*)-Rg_3_ and CK in the feces of ginseng polysaccharides group decreased about 52.41% and 40.46% (from 201318.50 ± 137314.20 ng/g to 95815.06 ± 55398.23 ng/g, and 789820.10 ± 190996.60 ng/g to 470279.30 ± 173142.50 ng/g), respectively (Fig. 5c).

Overall, the variation in the model group described above were driven primarily by significant decrease of *AUC* and/or *C*_max_ of secondary ginsenosides (20(*S*)-Rg_2_, Rd and 20(*S*)-Rg_3_) whereas significant increase of the fecal contents of primary ginsenosides (Re and Rc). However, ginseng polysaccharides treatment was able to turn this situation around. Therefore it was conductive to draw the conclusion that OACS weakened the intestinal metabolism and absorption of certain ginsenosides, which can be reversed by ginseng polysaccharides treatment.

## Discussion

Till now, dozens of polysaccharides in different structural types have been isolated and purified from the boiling water extracts (decoction) of ginseng, and their biological activities were preliminarily demonstrated^32^. These purified polysaccharides always possessed a low molecular weight less than 100 kDa. Interestingly, however, a recent study declared that high molecular weight polysaccharides (more than 100 kDa) played a biologically pivotal role in total ginseng polysaccharides^33^. These findings imply that bioactive polysaccharides in DST may cover a wide molecular weight distribution. Moreover, dietary prebiotics, from long-chain inulin to oligofructose, bear a very broad molecular weight range as well. Given these, and also in light of TCM wholism, this study focused total polysaccharides in DST with a very wide molecular weight distribution (1.00~1308.98 kDa). In agreement with previous studies^32^, multiple monosaccharides, i.e. Man, Rha, GalA, Glc, Gal, Ara and Fuc, constituted the total ginseng polysaccharides.

Ginsenosides can be categorized by either structural or metabolic properties^30^. According to structural properties, ginsenosides are normally classified into four types, namely protopanaxadiol, protopanaxatriol, ocotillol and oleanolic acid types. Among them, protopanaxadiol and protopanaxatriol types dominate; from the aspect of metabolic pathways, primary ginsenosides and secondary ginsenosides (metabolized from primary ginsenosides) are included. Taken together into consideration, the eleven ginsenosides aforementioned, including seven protopanaxadiol types (Rb_1_, Rc, Rb_2_, Rd, F_2_, 20(*S*)-Rg_3_, CK) and four protopanaxatriol types (Rg_1_, Re, 20(*S*)-Rg_2_, Rf) or, alternatively, seven primary ginsenosides (Re, Rf, Rb_1_, Rc, Rb_2_, Rg_1_, Rd) and four secondary ginsenosides (20(*S*)-Rg_2_, F_2_, 20(*S*)-Rg_3_, CK) were chosen as the representatives for investigation in this study. HPLC-TQ-MS with multiple reaction monitoring (MRM) scan was adopted for quantitative determination of the ginsenosides in the biological samples (plasma and feces) to provide the greatest selectivity and sensitivity. By five channels with different mass transitions, cone and collision voltages (Supplementary Table 6), the eleven analytes were detected with baseline separation (Fig. 3). The quantitative method for the biological samples was fully validated in terms of linearity, sensitivity, precision, accuracy, and stability tests (Supplementary Tables 7–9). Besides, UPLC-QTOF-MS was auxiliary for qualitative analysis by comparing the mass spectra and retention times with those of reference compounds and tentatively assigned by matching the empirical molecular formula with the published known ginsenosides, and/or further confirmed by elucidating the quasi-molecular ions and fragment ions in each group^34^.

According to the theory of traditional Chinese medicine, DST is clinically used for reinforcing *Qi* (vital energy). Forced swimming is a well-established model to imitate the *Qi*-deficiency status^35^. Moreover, it was previously defined that over-fatigue caused by forced swimming and acute cold stress may trigger a rapid loss of homeostasis of endogenous metabolism on rat models^36^. On the other side, functional interactions between gut microbiota and host endogenous metabolism has been well demonstrated^22^. Associating them, we presumed that OACS could elicit gut microbiota dysbiosis. Indeed, not only disorder of endogenous metabolism but also dysbiosis of gut microbiota caused by OACS were experimentally verified in this study. And their potential relations can be reasonably elucidated on the basis of previous studies. For examples, mammalian gut microbiota manages choline metabolism by transforming it into dimethylamines, trimethylamines and finally TMAO^37^. And the gut symbiotic bacterial metabolism has been defined as the exclusive pathway to produce TMAO from choline^38^. Likewise, gut microbiota regulates bile acid metabolism as well. CA and chenodeoxycholic acid, which are primary bile acids synthesized in the liver from cholesterol, can be metabolized by the gut microbiota into secondary bile acids^39^. Therefore the changed gut microorganism altered the urinary level of TMAO and the plasmatic level of CA in this study. The other gut microbiota derived metabolites, such as hippurate, 4-HPPA and 4-MP, also significantly varied in the model group^40^. Actually, besides microbial metabolism, it is worthy of attention that both choline and bile acids are strongly correlated with catabolism and/or anabolism. Specifically, bile acids serve as molecular modulators for glucose metabolism by binding to related cellular receptors^41^, while choline is implicated in lipid metabolism and synthesis of very-low-density lipoprotein^42^. It signifies that gut microbiota and host metabolism could functionally interact, which may result in gut microbiome and intermediary metabolites, such as citrate and isocitrate, are indirectly relevant. Dominant changes on the phylum Firmicutes and Bacteroidetes of gut microbiota have been found in metabolic syndromes with disordered endogenous metabolisms, such as obesity and type-2 diabetes, but the variations were inconsistent. A decrease of Firmicutes and an increase of Bacteroidetes were observed in some studies^43^, whereas the inverse was also reported^44^. Consistent with the latter, here Bacteroidetes was reduced and Firmicutes was enhanced in the model group by OACS.

In this study, ginseng polysaccharides imparted a well prebiotic-like effect to OACS rats by simultaneously stimulating the growth of two most important probiotics: *Lactobacillus* spp. and *Bacteroides* spp. More interestingly, beyond that, ginseng polysaccharides holistically restored the gut microflora perturbed by OACS. The mechanisms involved might be intricate. Because of very limited enzymes encoded in the human genome, most extraneous polysaccharides are indigestible until they reach the intestinal tract, where they can be fermented by gut microbe into short-chain fatty acids and lactate^45^. Gut microbial species vary in their polysaccharide preferences, and their strategies for competing polysaccharides are also different. Among them, the best elaborated mechanism is for members of phylum Bacteroidetes. They always possess various PULs (polysaccharide utilization loci)-encoded products, which are collectively termed Sus (starch utilization system)-like systems. These systems harbor several to decades of enzymes to degrade different polysaccharides by targeting specific glycosidic linkages or chemical substituents in the polysaccharides^46^. The mechanism for harvesting exogenous polysaccharides is common in Bacteroidetes members and is unique to this phylum^17^. Multiple natural polysaccharides such as pectin (homogalacturonan, rhamnogalacturonan I and II, etc.) and starch have been well demonstrated to be digested by Bacteroidetes with Sus-like systems^47^. Similar situation is very likely to occur on ginseng polysaccharides. Previously water-soluble polysaccharides from ginseng were systematically studied^48^. The results demonstrated the neutral portion of ginseng polysaccharides was a mixture of starch-like glucan and arabinogalactan while the acidic polysaccharides were identified as rhamnogalacturonan I-rich pectins and homogalacturonan-rich pectins. In agreement with it, an earlier report described that most fractions of ginseng polysaccharides were believed to be acidic pectic polysaccharides due to the dominant content of galacturonic acid^49^. Another two ginseng polysaccharides were elucidated as acidic arabinogalactans, and their backbones mainly consisted of (1 → 5)-*α*-_L_-Ara*f* and (1 → 3)-*β*-_D_-Gal*p*, occasionally branched at O-3 of Ara*f* or O-4 and O-6 of Gal*p*^50^. This structural feature is highly similar with several side chains of rhamnogalacturonan I containing individual, linear, or branched *α*-_L_-Ara*f* and *β*-_D_-Gal*p* residues^47^. Given all these facts, we extrapolate that Sus-like system driven degradation should be largely responsible for how ginseng polysaccharides affect phylum Bacteroidetes in the treated group. In addition, the polysaccharide acquisition strategies of other phylum are also preliminarily revealed, although they are still less well understood. For example, unlike Bacteroidetes, Firmicutes and Actinobacteria encode very few carbohydrate-degrading enzymes but possess abundant polysaccharide-specific ABC (ATP-binding cassette) transporters, by which degraded carbohydrates can be transported^51^. It provides a potential reason for variation of phylum Firmicutes and Actinobacteria shaped by ginseng polysaccharides. Except for the direct effects as prebiotics, it is worthy of special note that indirect effects of ginseng polysaccharides on gut microbiota of OACS rats potentially occurred as well. In this study, we clearly demonstrated that OACS significantly perturbed the composition of gut microbiota. On the other side, reportedly ginseng polysaccharides showed well bioactivities of anti-fatigue and anti-stress^52^,^53^, which were further confirmed on the OACS model in this study by the metabolomics analysis (Supplementary Figure 5), although the effective mechanisms are still unknown. Therefore modulation of systemic responses to the OACS by ginseng polysaccharides might suggest some other unclear mechanisms on its effects on gut microbiota that deserves further investigation.

Gut microbial metabolism of some primary ginsenosides investigated here has been well defined in previous reports, and also proven to be the main metabolic pathway *in vivo*. As summarized in Fig. 6, by selective deglycosylation, ginsenoside Re is metabolized by *Bacteroides* spp. to Rg_1_ and secondary ginsenosides 20(*S*)-Rg_2_^54^, while *Lactobacillus* spp. and *Bacteroides* spp. transform ginsenoside Rc into ginsenosides 20(*S*)-Rg_3_ and Rd, and then CK^55^,^56^, and that improved bioavailability and bioactivities of these secondary ginsenosides were also revealed therein. In this study, the relative abundances of *Lactobacillus* spp. and *Bacteroides* spp. in the model rats were decreased by OACS. Given their above-described roles, such variations would weaken intestinal metabolism and absorption of ginsenosides Re and Rc. As expected, the *C*_max_ of ginsenoside 20(*S*)-Rg_3_ and the *AUC* of ginsenosides 20(*S*)-Rg_2_, Rd, 20(*S*)-Rg_3_ were significantly decreased by OACS. Actually, the *C*_max_ of ginsenosides Rg_1_, Rd, 20(*S*)-Rg_2_ as well as the *AUC* of ginsenosides Rg_1_ and CK were also substantially reduced without significant differences. The results hinted that the intestinal metabolism of ginsenosides Re and Rc might be conspicuously weakened in the model group. The conclusion was further confirmed by the significant increase of ginsenosides Re and Rc in the feces by OACS. The excessive ginsenosides Re and Rc in the feces should be largely from the untransformed and/or unabsorbed prototypes in the intestinal tract. Meanwhile fecal contents of ginsenosides Rd and Rg_1_ was correspondingly reduced in the model group. However, the excreted amount of ginsenosides 20(*S*)-Rg_2_ in the feces was abnormally increased significantly, which might be attributed to permeability alternation of intestinal epithelial barrier caused by the disorder of gut microflora^57^.

Fecal chemical profiles were qualitatively consistent in the four groups, suggesting that metabolic pathways and mechanisms of ginsenosides in DST are not changed by either OACS or carbohydrate intervention. By the treatment of ginseng polysaccharides, the *C*_max_ of ginsenosides Rg_1_, Rd and 20(*S*)-Rg_3_ together with the *AUC* of ginsenosides 20(*S*)-Rg_2_, Rd and 20(*S*)-Rg_3_ were significantly increased compared with the model group. Concurrently the fecal contents of ginsenosides Re, Rc, 20(*S*)-Rg_2_, Rd, 20(*S*)-Rg_3_ and CK were significantly reduced in the ginseng polysaccharides group. These findings clearly demonstrated that intestinal biotransformation of ginsenosides Re and Rc was observably strengthened by ginseng polysaccharides. Further examinations showed ginseng polysaccharides stimulated the growth of *Lactobacillus* spp. and *Bacteroides* spp., and, more meaningfully, restored the entire gut microbiota perturbed by OACS. These correlated outcomes promote us to conclude that ginseng polysaccharides can enhance systemic exposure of certain ginsenosides *in vivo* in the OACS-induced rats *via* gut microbiota-mediated mechanisms. Similar results were also observed on oligofructose, but the variation degrees were not exactly alike with those by ginseng polysaccharides, which indicated that effects of different carbohydrates on gut microbiota and systemic exposure of ginsenosides may be selective. Actually, except for *Lactobacillus* spp. and *Bacteroides* spp., some other probiotics, such as *Bifidobacteria spp.*, were also involved in intestinal biotransformation of ginsenosides^58^. However, they were determined with very limited abundances (Others in Fig. 2b) or even not found in this study. Thus they were not specifically investigated here. In addition to Re and Rc, metabolism of other primary ginsenosides by intestinal microflora are also well documented. For example, ginsenosides Rb_1_ or Rb_2_ can be transformed into ginsenosides F_1_ or Rg_3_ by gut microbiota^59^,^60^. However, in this study, no significant change was determined in plasmatic and fecal contents of ginsenosides Rb_1_ and Rb_2_ between the four groups. In our previous study on chemical characterization of DST, we found that multiple malonyl-ginsenosides and acetyl-ginsenosides, e.g. malonyl-Rb_1_ and malonyl-Rb_2_, occurred in DST^26^. They could be degraded to their neutral ginsenosides by demalonylation or deacetylation, which would reach a dynamic equilibrium *in vivo* with the further degradation of the neutral ginsenosides. Such a dynamic equilibrium occurring on Rb_1_ and Rb_2_ (malonyl-Rb_1_ → Rb_1_ → Rg_3_/F_1_, etc.; malonyl-Rb_2_ → Rb_2_ → Rg_3_/F_1_, etc.) might be the reason of their seemingly invariable contents in the plasma and feces.

In summary, our findings provide a novel gut microbiota-involved mechanism by which polysaccharides synergistically work with small molecular chemicals co-existed in TCM decoction on certain pathological model. The facts inspire that TCM polysaccharides, even indigestible by host, could still indirectly contribute to therapeutic effects of TCM decoction. By conferring TCM polysaccharides a new role, this study is instrumental in scientization and rationalization of the classic TCM decoctions in human health care, and therefore is meaningful for inheritance and innovation of TCM decoctions.

## Materials and Methods

### Chemicals and materials

HPLC grade acetonitrile and methanol were purchased from Tedia Co., INC. (Fairfield, USA). MS-grade formic acid was supplied by ROE Scientific INC Co. (Dover, DE, USA). Ultra-pure water was produced by a Milli-Q water purification system (Milford, MA, USA).

**Figure 1:**
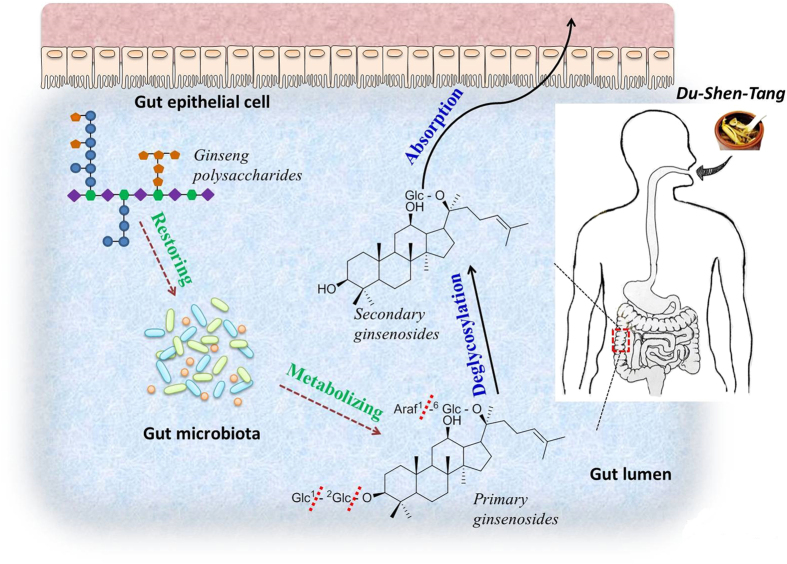
Extrapolated gut microbiota involved relation between ginseng polysaccharides and ginsenosides co-administered in DST.

**Figure 2:**
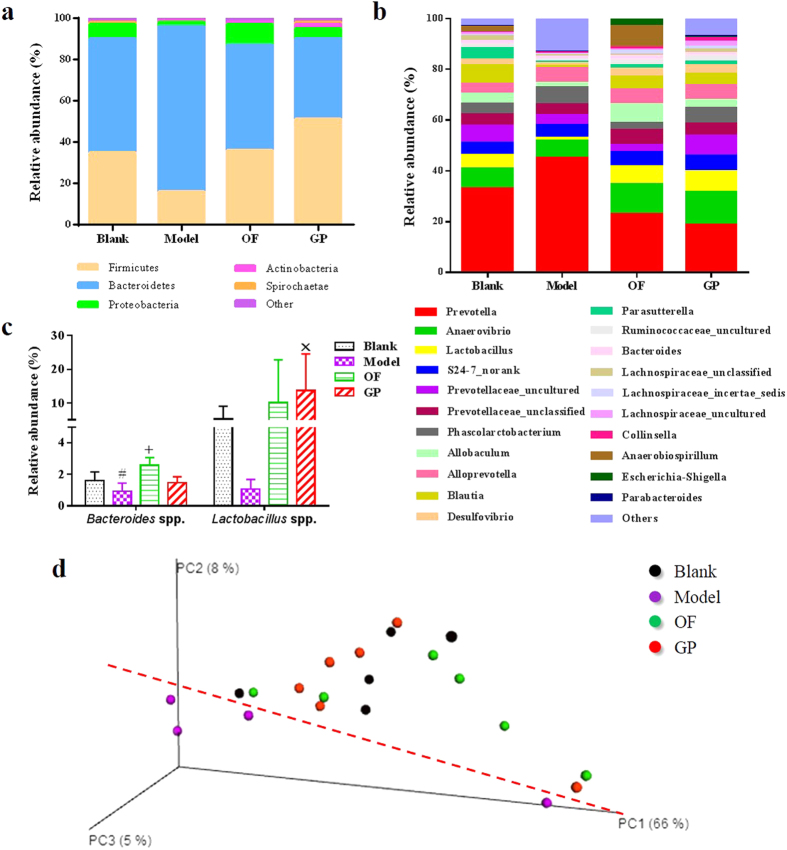
Comparison of gut microbiota in the four group rats by the relative abundances of phylum (a), genus (b), *Bacteroides* spp. and *Lactobacillus* spp. (**c**), and by PCoA (**d**) (n = 6). Blank: the blank group; Model: the model group; OF: the oligofructose group; GP: the ginseng polysaccharide group (the same below); (**#**: 0.05 < *p* < 0.10, compared with Blank; ^+^*p* < 0.05, compared with Model; ×: 0.05 < *p* < 0.10, compared with Model).

**Figure 3:**
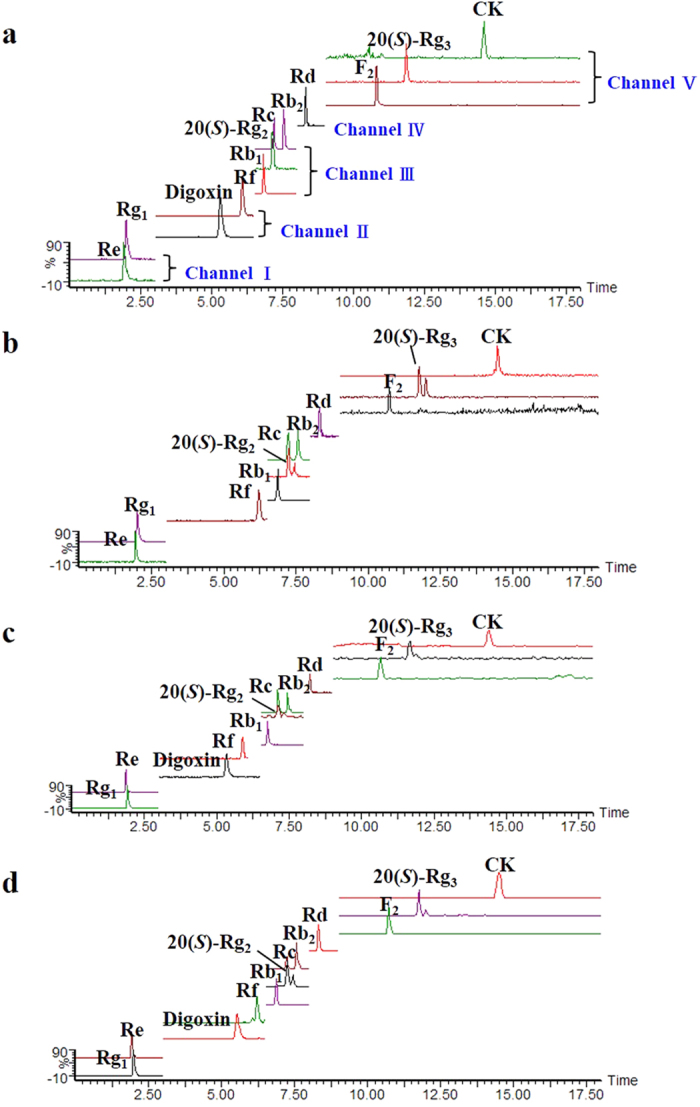
Base peak ion chromatograms of the ginsenoside mixed standards (a), herbal extracts (b), plasma (c) and feces (d) by HPLC-TQ-MS analysis with MRM scan (in positive ion mode).

**Figure 4:**
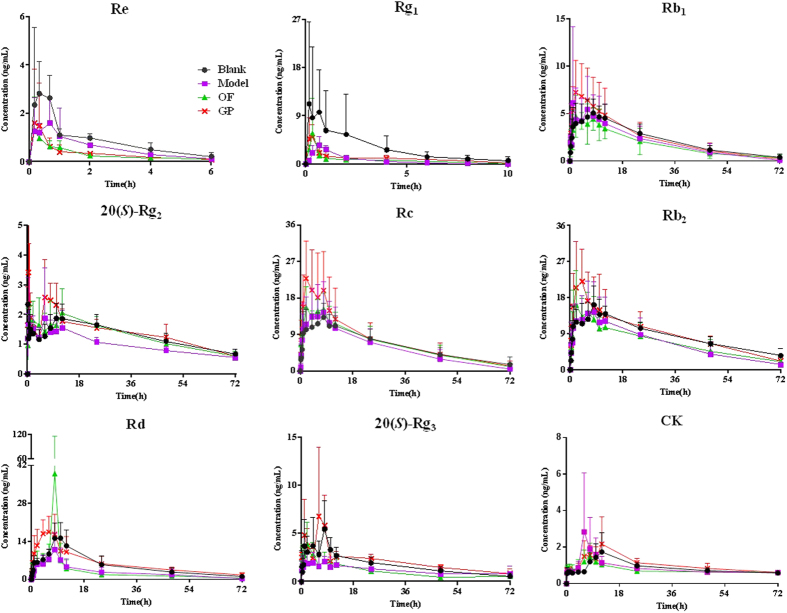
Mean plasma concentration-time profiles of nine ginsenosides in the four group rats after i.g. administration of ginsenoside extracts (n = 6).

**Figure 5:**
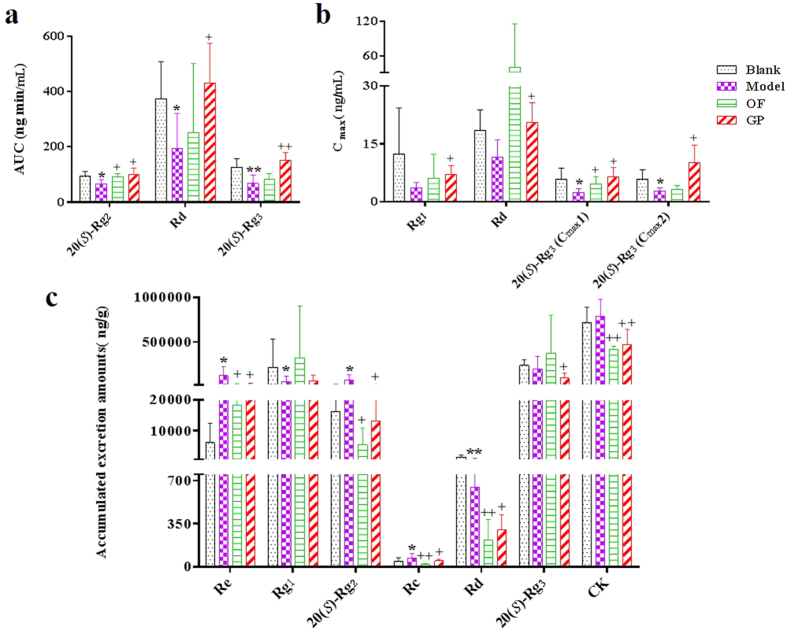
Comparison of *AUC* (**a**), *C*_max_ (**b**) and 24 h accumulated excretion amounts (**c**) of certain ginsenosides in the four group rats after i.g. administration of ginsenoside extracts (n = 6); (*p < 0.05, **p < 0.01, compared with Blank; ^+^*p* < 0.05, ^**+**+^*p* < 0.01, compared with Model).

**Figure 6:**
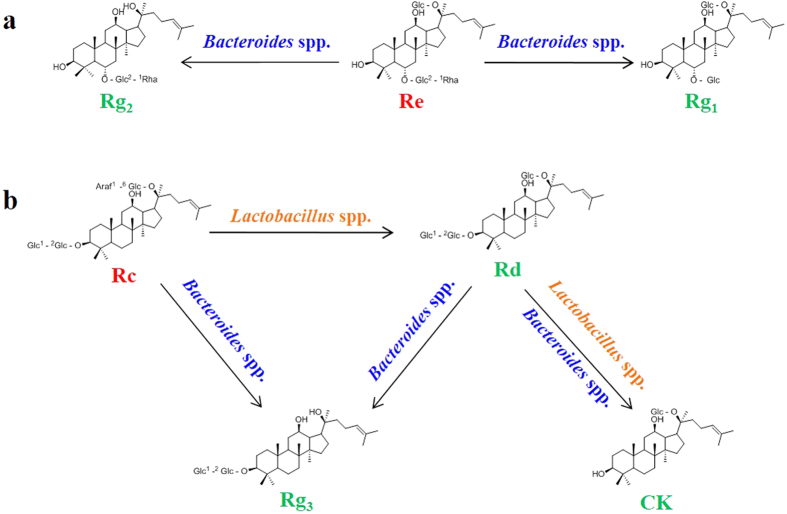
Gut microbial metabolisms of ginsenosides Re (a) and Rc (b) by B*acteroides* spp. and/or *Lactobacillus* spp.

Ginsenosides Rg_1_ (**1**), Re (**2**), Rf (**3**), pseudoginsenoside F_11_ (Pseudo-F_11_) (**4**), pseudoginsenoside Rt_5_ (Pseudo-Rt_5_) (**5**), 20(*R*)-notoginsenoside R_2_ (*R*-Note-R_2_) (**6**), 20(*S*)-Rg_2_ (**7**), Rb_1_ (**8**), 20(*S*)-Rh_1_ (**9**), F_3_ (**10**), 20(*R*)-Rg_2_ (**11**), 20(*R*)-Rh_1_ (**12**), Rc (**13**), Ro (**14**), Rb_2_ (**15**), Rb_3_ (**16**), F_1_ (**17**), Rd (**18**), F_2_ (**19**), notoginsenoside Ft_1_ (Note-Ft_1_) (**20**), 20(*S*)-Rg_3_ (**21**), 20(*S*)-protopanaxatriol (20(*S*)-PPT) (**22**), Compound K (CK) (**23**), 20(*S*)-Rh_2_ (**24**), 20(*R*)-Rh_2_ (**25**), Rh_3_ (**26**) and Digoxin were purchased from Chengdu Munster Biotechnology Co. Ltd. (Chengdu, China), and their structures are shown in Fig. 1. The purity of these ginsenosides was higher than 95.0% by HPLC analysis. Oligofructose (OF) was provided by BENEO GmbH (Mannheim, Germany). A series of Dextrans with different molecular weights, 670 kD, 410 kD,270 kD, 150 kD, 80 kD, 50 kD, 25 kD, 12 kD, 5 kD, 1 kD and D-galacturonic acid monohydrate (GalA), D-glucuronic acid (GlcA), L-arabinose (Ara), D-mannose (Man), D-galactose (Gal), D-glucose (Glc), L-rhamnose monohydrate (Rha), D-fucose (Fuc), D-ribose (Rib) were purchased from Sigma (St. Louis, MO, USA).

Fresh ginseng was collected from Jilin Province of China. The samples were authenticated by Prof. S.L. Li to be the root of *P. ginseng* based on morphological and histological features according to the standards of Chinese Pharmacopoeia (2010 version). The voucher specimens were deposited in Department of Pharmaceutical Analysis and Metabolomics, Jiangsu Province Academy of Traditional Chinese Medicine.

### DST, ginsenosides and ginseng polysaccharides preparation and characterization

6 kg of dried ginseng slices were refluxed with 14-fold distilled water at 100 °C for 1.5 h. The extraction were repeated twice, the extracted solutions were filtered and then combined to generate DST.

DST were concentrated and precipitated with 95% ethanol. The generated precipitate was then washed with Sevag reagent (isoamyl alcohol and CHCl_3_ in 1:4 ratio) and dried under vacuum, yielding the crude polysaccharides (the polysaccharide content was higher than 95.0% by compositional monosaccharide analysis).

The supernatants by ethanol precipitation were vacuum concentrated and freeze-dried, and then refluxed with 14-fold 75% ethanol for 1.5 h. The extracts were combined and then evaporated vacuum to obtain ginsenosides extract.

The prepared ginseng polysaccharides were chemically characterized in terms of molecular weight distribution and monosaccharide composition analysis by using our previous methods^31^.

### Animal experiments

Male Sprague-Dawley rats (200 ± 20 g) were purchased from the Shanghai Laboratory Animal Co. (SCXK, Shanghai, China) (fully accredited by the Association for the Assessment and Accreditation of Laboratory Animal Care International). Each animal was evaluated to be in good health, and then acclimated to the laboratory environment (12 h light/dark cycle, at 23–27 °C, and 30–60% relative humidity) for one week before experiment. Feed and potable water were provided *ad libitum* during molding and were stopped in the last two days of experiments. All the animal experimental protocols were approved by the Animal Care Ethics Committee, Jiangsu Province Academy of Euthanasia for Animals, and the methods were carried out in accordance with Administrative Measures of Experimental Animals in Jiangsu Province.

Twenty-four male Sprague-Dawley rats were randomly divided into four groups of six rats each, blank group, model group, oligofructose group and ginseng polysaccharides group. Rats in the model group, oligofructose group and ginseng polysaccharides group were forced loaned-swimming by hanging a clog (5% of the rat body weight) on the tail in a self-manufactured swimming pool. The rats swam twice each day with an interval of ten min for fourteen consecutive days. The water temperature was maintained at 25 ± 2 °C at the first thirteen days, but 0 ± 2 °C on the last day. In the meantime, rats in the blank were free without any forced activities. During the fourteen days, the blank and model groups were daily i.g. administered saline (200 mg/kg), while oligofructose group and ginseng polysaccharides group were daily i.g. administered oligofructose (200 mg/kg) and ginseng polysaccharides extracts (200 mg/kg), respectively. On the last day (the fourteenth day), all the rats in the four groups were i.g. administered ginsenosides solution (500 mg/kg) after swimming, and then they were placed into separate metabolic cages for biological sample collection.

### Biological sample collection and treatment

Blood samples (approximately 200 μL) were collected from the retinal vein plexus into test tubes containing sodium heparin at 0, 0.16, 0.33, 0.67, 1, 2, 3, 4, 6, 8, 10, 12, 24, 48, 72 h after the ginsenosides administration. Plasma was separated and prepared by centrifugation of the blood sample at 5000 rpm for 5 min, and then stored at −20 °C for further analysis. 0-h fresh feces, 24-h feces and urine sample were collected and stored at −80 °C until analysis.

### 16S rRNA gene sequence analysis

Genomic DNA was extracted by an InviMag Stool DNA Kit (Invitek, Berlin, Germany) from the 0-h fresh feces. The V3-V4 region of the bacteria 16S ribosomal RNA gene were amplified by PCR (95 °C for 3 mins, followed by 27 cycles of 95 °C for 30 s, 55 °C for 30 s, 72 °C for 45 s, and 72 °C for 10 mins) using primers 338F (5′-ACTCCTACGGGAGGCAGCA-3′) and 806R (5′-GGACTACHVGGGTWTCTAAT-3′), where barcode is an eight-base sequence unique to each sample. PCR reactions were performed in triplicate 20 μL mixture containing 4 μL of 5 × FastPfu Buffer, 2 μL of 2.5 mM dNTPs, 0.8 μL of each primer (5 μM), 0.4 μL of FastPfu Polymerase, and 10 ng of template DNA. Amplicons were extracted from 2% agarose gels and purified using the AxyPrep DNA Gel Extraction Kit (Axygen Biosciences, Union City, CA, U.S.) according to the manufacturer’s instructions and quantified using QuantiFluor™ -ST (Promega, U.S.). Purified amplicons were pooled in equimolar and paired-end sequenced (2 × 250) on an Illumina MiSeq platform according to the standard protocols. The raw reads were deposited into the NCBI Sequence Read Archive (SRA) database. Raw fastq files were demultiplexed, quality-filtered using QIIME (version 1.17) with the following criteria: (i) The 300 bp reads were truncated at any site receiving an average quality score <20 over a 50 bp sliding window, discarding the truncated reads that were shorter than 50bp. (ii) exact barcode matching, 2 nucleotide mismatch in primer matching, reads containing ambiguous characters were removed. (iii) only sequences that overlap longer than 10 bp were assembled according to their overlap sequence. Reads which could not be assembled were discarded. Operational Units (OTUs) were clustered with 97% similarity cutoff using UPARSE (version 7.1) and chimeric sequences were identified and removed using UCHIME. The taxonomy of each 16S rRNA gene sequence was analyzed by RDP Classifier (http://rdp.cme.msu.edu/) against the silva (SSU115)16S rRNA database using confidence threshold of 70%.

### Metabolomics by UPLC-QTOF-MS

The 0-h plasma and urine samples were thawed at room temperature, and then treated in the same way as follows. The sample (200 μL) was mixed with 800 μL methanol, and the mixture was centrifuged at 10000 rpm for 10 min at 4 °C. The supernatant was transferred and evaporated to dryness at 45 °C under a gentle stream of air. The residue was dissolved with 200 μL methanol solution. After centrifugation again at 10000 rpm for 10 min, the supernatant was collected for UPLC-QTOF-MS analysis.

UPLC was performed on a Waters ACQUITY UPLCTM system (Waters Corporation, Milford, MA, USA) equipped with a binary solvent delivery system and an auto-sampler. The chromatographic separation was achieved with a Waters ACQUITY HSS T3 (2.1 mm × 100 mm, I.D., 1.8 μm). The mobile phase consisted of (A) 0.1% formic acid in water and (B) acetonitrile containing 0.1% formic acid. The elution condition was optimized as follows: 5% B (0–2 min), 5–50% B (2–8 min), 50–95% B (8–8.2 min), 95% B (8.2–11 min), 95–5% B (11–12 min) and isocratic at 5% B (12–13 min). The flow rate was 0.4 mL/min. The column and auto-sampler were maintained at 35 °C and 10 °C, respectively. The injection volume was 1 μL.

Mass spectrometry was performed on a Waters SYNAPT G2-S QTOF system (Waters MS Technologies, Manchester, UK) coupled with electrospray ionization (ESI) interface. The mass spectrometer was operated in both negative and positive ion modes. The desolvation gas was set to 800 L/h at 450 °C. The cone gas was 40 L/h. The source temperature was 100 °C. The capillary voltage and cone voltage were set at 3000 V and 35 V, respectively. The QTOF acquisition rate was 0.2 s. The energies for collision-induced dissociation (CID) were set at 4 eV and 20–30 eV respectively for the fragmentation information. Centroided data were acquired for each sample over a mass range of m/z 50–900 with dynamic range enhancement (DRETM) applied throughout the MS experiment to ensure accurate mass measurements.

### Pharmacokinetics by HPLC-TQ-MS

The plasma samples during 0–72 h were used for pharmacokinetic study. 200 μL plasma sample and 40 μL Digoxin (5 μg/mL) were mixed with 800 μL of methanol. The mixture was then vortex-extracted for 5 min, and centrifuged at 12000 rpm for 10 min at 4 °C. The supernatant was transferred and evaporated to dryness at 45 °C in a rotary evaporator. The residue was dissolved with 200 μL methanol solution. After centrifugation again at 12000 rpm for 10 min, the supernatant was collected for HPLC-TQ-MS analysis.

The HPLC analysis was performed on a Waters Alliance HPLC 2695 system (Waters Corp., MA, USA), equipped with a binary solvent delivery system and an auto-sampler. The chromatographic separation was achieved on an Agilent Poroshell 120 EC-C_18_ column (100 mm × 3.0 mm, 2.7 μm) with a Phenomenex C_18_ guard column. The mobile phase consisted of (A) 1 μM sodium formate aqueous solution and (B) acetonitrile. The gradient elution was optimized as follow: 28–30% B (0–2 min), 30–60% B (2–10 min), 60–80% B (10–12 min), 80–95% B (12–14 min), 95–28% B (14–16 min), 28% B (16–19 min). The flow rate was 0.4 mL/min. The column and auto-sampler temperature were maintained at 35 °C and 10 °C, respectively. The injection volume was 10 μL.

Mass spectrometry was performed on a Micromass Quattro-Micro™ triple-quadrupole mass spectrometer (Waters Co., Milford, MA, USA) with electrospray ionization (ESI) interface in negative mode. For all the mass scan modes, the capillary voltage was 3,500 V, desolvation gas was set to 450 L/h at 400 °C, and source temperature was set at 120 °C. Multiple reaction mode (MRM) was employed for analysis. Details of monitoring conditions for each analytes were summarized in Supplementary Table 3. Argon was employed as the collision gas at a pressure of 4.0 × 10^−3^ mbar. Method validation for quantitative analysis was fully performed.

### Feces analysis by UPLC-QTOF-MS and HPLC-TQ-MS

0.05 g 24-h feces sample mixed with 200 μL Digoxin (5μg/mL) were extracted by ultra-sonication with 1 mL methanol for 30 min, the extracted solution was centrifuged at 12000 rpm for 10 min at 4 °C. Then the supernatant was evaporated to dryness at 45 °C in a rotary evaporator. The residue was dissolved with 1 mL methanol solution. After centrifugation again at 12000 rpm for 10 min, the supernatant was collected for UPLC-QTOF-MS and HPLC-TQ-MS analysis, respectively.

The UPLC-QTOF-MS elution condition was optimized as follows: 5–15% B (0–1 min), 15–60% B (1–22 min), 60–95% B (22–23 min), 95% B (23–24 min), 95–5% B (24–26 min) and isocratic at 5% B (26–27 min). The Q-TOF mass spectrometer was operated in negative ion modes. The desolvation gas was set to 900 L/h at 450 °C. The capillary voltage and cone voltage were set at 2500 V and 30 V, respectively. The energies for collision-induced dissociation (CID) were set at 5 eV and 45 eV, respectively, for fragmentation information. Centroided data were acquired for each sample from 100–1500 Da. Other unmentioned conditions in UPLC-QTOF-MS were same with metabonomics analysis. The HPLC-TQ-MS analysis was performed by reference to the pharmacokinetic study.

### Statistical analysis

Statistical analysis was performed using Prism software (Graphpad). Data was plotted in the figures as mean ± SEM. Differences between two groups were assessed using two-tailed, unpaired Student’s t test with Welch’s correction. Significant differences were indicated in the figures by * or ^+^*p* < 0.05, ** or ^++^*p* < 0.01. Notable near-significant differences (0.05 < *p* < 0.1) were indicated in the figures by # or ×. Notable nonsignificant (and non-near significant) differences were not indicated.

The pharmacokinetic parameters of ginsenosides were analyzed by a non-compartmental method using the nonlinear least squares regression program BAPP (version 2.0 PK, Chinese Pharmaceutical University). The peak plasma concentration (*C*_max_) and the time to reach *C*_max_ (*T*_max_) after oral administration were obtained. The area under the concentration-time curve from *0* to *t* h *(AUC*_0-t_) and the terminal elimination half-life (*t*_1/2_) were calculated by the following equations 1 and 2:


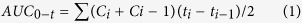



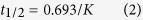


Here *K* means the terminal rate constants.

## Additional Information

**How to cite this article**: Zhou, S.-S. *et al.* Gut microbiota-involved mechanisms in enhancing systemic exposure of ginsenosides by coexisting polysaccharides in ginseng decoction. *Sci. Rep.* **6**, 22474; doi: 10.1038/srep22474 (2016).

## Supplementary Material

Supplementary Information
