# Supplementary material for: Gut microbiota-involved mechanisms in enhancing systemic exposure of ginsenosides by coexisting polysaccharides in ginseng decoction
Source: Sci Rep. 2016 Mar 2;6:22474. doi: 10.1038/srep22474 (PMC4774164; doi:10.1038/srep22474)
Supplement: Supplementary Information [file srep22474-s1.pdf]

Gut microbiota-involved mechanisms in enhancing systemic exposure of ginsenosides by coexisting polysaccharides in ginseng decoction

Shan-Shan Zhou[1, 2, #], Jun Xu[3, #], He Zhu[1], Jie Wu[2], Jin-Di Xu[1], Ru Yan[4], Xiu-Yang Li[1, 2], Huan-Huan Liu[1, 2], Su-Min Duan[2], Zhuo Wang[2], Hu-Biao Chen[3], Hong Shen[2, *], Song-Lin Li[1, 2, *]

Supplementary Table 1 Linearity, sensitivity and contents of the eleven ginsenosides in DST (*n*=3)

Analyte	Linear range (ng/mL)	Equation	R^2	LOD (ng/mL)	LOQ (ng/mL)	Contents (μg/mL)
Re	1.00-200	y=5.23x+29.63	0.9990	0.50	1.00	225.70 ± 5.10
Rg_1	1.00-200	y=7.15x+239.04	0.9991	0.50	1.00	360.69 ± 17.53
Rf	1.00-200	y=19.28x+51.01	0.9996	0.50	1.00	107.72 ± 2.35
Rb_1	1.00-200	y=942.97x+5804.09	0.9990	0.50	1.00	52.01 ± 1.18
20(*S*)-Rg_2	1.00-200	y=2.06x+7.10	0.9992	0.50	1.00	83.83 ± 2.58
Rc	1.00-200	y=33.13x+503.00	0.9990	0.50	1.00	190.51 ± 9.18
Rb_2	1.00-200	y=42.02x+537.50	0.9991	0.50	1.00	198.54 ± 7.87
Rd	1.00-200	y=2.84x+1.95	0.9992	0.50	1.00	72.36 ± 2.82
F_2	1.00-200	y=20.49x+31.73	0.9998	0.50	1.00	1.08 ± 0.13
20(*S*)-Rg_3	5.00-200	y=10.12x+11.24	0.9999	2.50	5.00	138.48 ± 6.85
CK	1.00-200	y=84.04x+349.78	0.9991	0.50	1.00	1.66 ± 0.03

Supplementary Table 2 Plasmatic and urinary biomarkers for the OACS model

Samples	Ion (*m/z*)	ESI mode	Biomarker identification	Molecular formula
Plasma	120.0818	+	Phenylalanine	$C_9H_{11}NO_2$
	232.1544	+	Butyrylcarnitine	$C_{11}H_{21}NO_4$
	188.0718	+	Tryptophan	$C_{11}H_{12}N_2O_2$
	355.2632	+	CA	$C_{24}H_{40}O_5$
	496.3409	+	LPC 16:0	$C_{24}H_{50}NO_7P$
	522.3562	+	LPC 18:1	$C_{26}H_{52}NO_7P$
	524.3725	+	LPC 18:0	$C_{26}H_{54}NO_7P$
	187.0073	-	Cresol sulfate	$C_7H_8O_4S$
Urine	188.99	-	Citrate	$C_6H_5O_7$
	188.99	-	Isocitrate	$C_6H_5O_7$
	151.04	-	4-hydroxyphenylacetate	$C_8H_7O_3$
	178..05	-	Hippurate	$C_9H_9NO_3$
	107.05	-	4-methyl-phenol	C_7H_8O
	76.04	+	Trimethylamine-N-oxide	C_3H_9NO

Supplementary Table 3 Main pharmacokinetics parameters of the eleven ginsenosides in the four group rats after i.g. administration of ginsenoside extracts (n=6)

Parameter	Re	Rg_1	Rb_1	20(*S*)-Rg_2	Rc	Rb_2	Rd	20(*S*)-Rg_3	CK
**C_{max} (ng/mL)**									
Blank	3.71 ± 0.82	12.33 ± 12.05	5.66 ± 1.37	3.33 ± 3.12 [a]	15.54 ± 2.78	17.52 ±3.76	18.52 ± 5.31	5.87 ± 2.87	1.90 ± 0.99
				2.20 ± 0.35 [b]				5.77 ± 2.56	
Model	2.22 ± 1.08	3.66 ±1.37	5.68 ±3.38	2.29 ± 1.27	16.67 ± 7.27	17.44 ± 6.90	11.67 ± 4.46	2.44 ± 0.96*	3.21 ± 2.97
				2.73 ± 1.12				2.75 ± 0.87*	
OF	1.74 ± 0.99	6.03 ±6.30	5.48 ± 1.60	2.06 ± 0.59	18.03 ± 4.22	18.54 ± 8.25	40.23 ± 76.03	4.71 ± 1.83[+]	1.72 ± 0.64
				2.44 ± 0.67				3.20 ± 1.07	
GP	2.01 ± 2.00	7.11 ±2.30[+]	7.24 ± 3.38	3.62 ± 2.84	24.94 ± 8.34	24.09 ± 10.00	20.54 ± 5.13[+]	6.36 ± 2.51[+]	2.49 ± 1.37
				3.30 ± 0.61				10.16 ± 4.56[+]	
**T_{max} (h)**									
Blank	0.39 ± 0.26	0.42 ± 0.29	6.50 ± 3.56	2.17 ± 1.47	6.17 ± 4.12	7.50 ± 3.56	9.00 ± 1.67	2.20 ± 1.64	10.33 ± 1.97
				8.67 ± 1.63				8.33 ± 0.82	
Model	0.39 ± 0.26	0.84 ± 0.19	8.00 ± 2.45	1.83 ± 1.17	6.67 ± 2.42	5.67 ± 2.94	6.80 ± 2.68	2.33 ± 1.37	8.67 ± 2.42
				8.33 ± 2.34				8.33 ± 2.34	
OF	0.28 ± 0.09	0.28 ± 0.09	2.93 ± 2.89	1.53 ± 0.65	4.40 ± 3.29	4.40 ± 2.61	4.40 ± 2.61	1.53 ± 0.65	8.80 ± 2.28
				7.60 ± 3.58				7.20 ± 3.35	
GP	0.34 ± 0.24	0.25 ± 0.09	2.00 ± 0.00	1.50 ± 0.58	5.00 ± 3.46	3.00 ± 1.16	5.00 ± 2.58	1.75 ± 0.96	9.50 ± 3.00

				7.00 ± 1.16				7.00 ± 1.16	
**t $_{1/2}$ (h)**									
Blank	1.88 ± 0.69	4.24 ± 2.31	20.08 ± 8.23	36.75 ± 3.55	24.92 ± 15.37	31.91 ± 12.46	20.19 ± 6.60	24.03 ± 9.62	49.393 ± 15.64
Model	2.44 ± 1.42	3.25 ± 2.48	18.35 ± 12.64	38.65 ± 4.19	15.76 ± 4.98	19.41 ± 10.48	10.76 ± 6.71	26.18 ± 6.80	45.75 ± 24.55
OF	3.69 ± 1.77	1.95 ± 0.89	15.81 ± 5.52	34.74 ± 2.69	19.54 ± 6.15	20.10 ± 8.62	18.26 ± 11.43	16.35 ± 10.62	20.90 ± 12.86
GP	2.51 ± 1.34	1.60 ± 0.56	12.99 ± 4.68	35.73 ± 1.86	17.70 ± 8.43	21.45 ± 3.91	26.63 ± 3.78	30.37 ± 10.86	52.63 ± 14.447
**AUC (ng·min/mL)**									
Blank	5.33 ± 1.35	29.79 ±32.46	161.22 ± 48.58	93.36 ± 17.58	447.35 ± 119.98	621.22 ± 114.03	374.62 ± 133.91	126.66 ± 31.23	63.43 ± 11.73
Model	3.04 ±1.37	7.36 ± 1.84	134.45 ± 92.45	65.66 ± 16.00*	414.01 ± 231.79	464.84 ± 239.85	195.27 ± 126.23*	67.47 ± 30.60**	57.68 ± 25.39
OF	1.89 ± 0.41	7.22 ± 2.20	133.56 ± 50.60	91.70 ± 11.65^{+}	468.52 ± 130.70	497.09 ± 181.79	251.32 ± 249.90	81.52 ± 21.74	57.45 ± 12.78
GP	2.19 ± 0.89	10.54 ± 4.31	172.70 ± 97.54	100.36 ± 23.21^{+}	542.29 ± 280.07	659.09 ± 236.66	431.74 ± 143.71^{+}	152.32 ± 27.23^{++}	75.99 ± 24.84

[a]: The value of the first peak;

[b]: The value of the second peak;

*: p<0.05, **: p<0.01, compared with Blank;

$^{+}$: p<0.05, $^{++}$: p<0.01, compared with Model.

Supplementary Table 4 Ginsenosides and relevant metabolites identified in the 24 h feces of the four group rats

Name	t_R (time)	Molecular formula	Characteristic ions
Rg_1	7.12	$C_{42}H_{72}O_{14}$	845.4915 [M-H+HCOOH]$^-$ (845.4899, 1.9); 799.4845 [M-H]$^-$ (799.4844, 0.1); 637.4335 [M-H-(Glc-H_2O)]$^-$ (637.4316, 3.0); 475.3773 [M-H-2(Glc-H_2O)]$^-$ (475.3787, -2.9)
Re	7.13	$C_{48}H_{82}O_{18}$	991.5497 [M-H+HCOOH]$^-$(991.5478, 1.9); 945.5436 [M-H]$^-$ (945.5423, 1.4); 799.4835 [M-H-(Rha-H_2O)]$^-$ (799.4844, -1.1); 783.4887 [M-H-(Glc-H_2O)]$^-$ (783.4895, -1.0); 637.4308 [M-H-(Rha-H_2O)-(Glc-H_2O)]$^-$ (637.4316, -1.3); 475.3790 [M-H-(Rha-H_2O)-2(Glc-H_2O)]$^-$ (475.3787, 0.6)
Rf	10.25	$C_{42}H_{72}O_{14}$	845.4920 [M-H+HCOOH]$^-$ (845.4899, 2.5); 799.4851 [M-H]$^-$ (799.4844, 0.9); 637.4315 [M-H-(Glc-H_2O)]$^-$ (637.4316, -0.2); 475.3781 [M-H-2(Glc-H_2O)]$^-$ (475.3787, -1.3)
20(*S*)-Rg_2	11.34	$C_{42}H_{72}O_{13}$	829.4954 [M-H+HCOOH]$^-$ (829.4949, 0.6); 783.4888 [M-H]$^-$ (783.4895, -0.9); 637.4324 [M-H-(Rha-H_2O)]$^-$ (637.4316, 1.3); 475.3786 [M-H-(Rha-H_2O)-(Glc-H_2O)]$^-$ (475.3787, -0.2)
Rb_1	11.46	$C_{54}H_{92}O_{23}$	1153.6012[M-H+HCOOH]$^-$ (1153.6006, 0.5); 1107.5961[M-H]$^-$ (1107.5951, 0.9)
20(*S*)-Rh_1	11.48	$C_{36}H_{62}O_9$	683.4373 [M-H+HCOOH]$^-$ (683.4370, 0.4); 637.4316 [M-H]$^-$ (637.4316, 0.0); 475.3793 [M-H-(Glc-H_2O)]$^-$ (475.3787, 1.3)
20(*R*)-Rg_2	11.53	$C_{42}H_{72}O_{13}$	829.4957 [M-H+HCOOH]$^-$ (829.4949, 1.0); 783.4899 [M-H]$^-$ (783.4895, 0.5); 637.4315 [M-H-(Rha-H_2O)]$^-$ (637.4316, -0.2); 475.3795 [M-H-(Rha-H_2O)-(Glc-H_2O)]$^-$ (475.3787, 1.7)
20(*R*)-Rh_1	11.86	$C_{36}H_{62}O_9$	683.4370 [M-H+HCOOH]$^-$ (683.4370, 0.0); 637.4299 [M-H]$^-$ (637.4316, -2.7); 475.3783 [M-H-(Glc-H_2O)]$^-$ (475.3787, -0.8)
Rc	11.90	$C_{53}H_{90}O_{22}$	1123.5905 [M-H+HCOOH]$^-$ (1123.5900, 0.4); 1077.5853 [M-H]$^-$ (1077.5845, 0.7); 945.5422 [M-H-(Ara(f)-H_2O)]$^-$ (945.5423, -0.1)

Ro	11.93	$C_{48}H_{76}O_{19}$	955.4907 [M-H]$^-$ (955.4903, 0.4); 793.4382 [M-H-(Glc-H_2O)]$^-$ (793.4374, 1.0)
Rb_2	12.33	$C_{53}H_{90}O_{22}$	1123.5901 [M-H+HCOOH]$^-$ (1123.5900, 0.1); 1077.5854 [M-H]$^-$ (1077.5845, 0.8); 945.5423 [M-H-(Ara(f)-H_2O)]$^-$ (945.5423, 0.0)
Rb_3	12.46	$C_{53}H_{90}O_{22}$	1123.5894 [M-H+HCOOH]$^-$ (1123.5900, -0.5); 1077.5845 [M-H]$^-$ (1077.5845, 0.0); 945.5416 [M-H-(Ara(f)-H_2O)]$^-$ (945.5423, -0.7)
F_1	12.88	$C_{36}H_{62}O_9$	683.4368 [M-H+HCOOH]$^-$ (683.4370, -0.3); 637.4319 [M-H]$^-$ (637.4316, 0.5); 475.3788 [M-H-(Glc-H_2O)]$^-$ (437.3787, 0.2)
Rd	13.30	$C_{48}H_{82}O_{18}$	991.5471 [M-H+HCOOH]$^-$(991.5478, -0.7); 945.5400 [M-H]$^-$ (945.5423, -2.4); 799.4835 [M-H-(Rha-H_2O)]$^-$ (799.4844, -1.1); 783.4889 [M-H-(Glc-H_2O)]$^-$ (783.4895, -0.8); 621.4360 [M-H-2(Glc-H_2O)]$^-$ (621.4366, -1.0)
F_2	16.65	$C_{42}H_{72}O_{13}$	829.4960 [M-H+HCOOH]$^-$ (829.4949, 1.3); 783.4891 [M-H]$^-$ (783.4895, -0.5); 621.4369 [M-H-(Glc-H_2O)]$^-$ (621.4366, 0.5); 459.3834 [M-H-2(Glc-H_2O)]$^-$ (459.3838, -0.9)
20(*S*)-Rg_3	17.68	$C_{42}H_{72}O_{13}$	829.4953 [M-H+HCOOH]$^-$ (829.4949, 0.5); 783.4897 [M-H]$^-$ (783.4895, 0.3); 621.4363 [M-H-(Glc-H_2O)]$^-$ (621.4366, -0.5); 459.3831 [M-H-2(Glc-H_2O)]$^-$ (459.3838, -1.5)
20(*S*)-PPT	18.67	$C_{30}H_{52}O_4$	521.3840 [M-H+HCOOH]$^-$ (521.3842, -0.4)
CK	21.92	$C_{36}H_{62}O_8$	667.4415 [M-H+HCOOH]$^-$ (667.4421, -0.9); 621.4352 [M-H]$^-$ (621.4366, -2.3); 459.3826 [M-H-(Glc-H_2O)]$^-$ (459.3838, -2.6)
20(*S*)-Rh_2	22.42	$C_{36}H_{62}O_8$	667.4427 [M-H+HCOOH]$^-$ (667.4421, 0.9); 621.4382 [M-H]$^-$ (621.4366, 2.6)
20(*R*)-Rh_2	22.76	$C_{36}H_{62}O_8$	667.4425 [M-H+HCOOH]$^-$ (667.4421, 0.6); 621.4351 [M-H]$^-$ (621.4366, -2.4)
Rg_6	15.64	$C_{42}H_{70}O_{12}$	811.4838 [M-H+HCOOH]$^-$ (811.4844, -0.7); 765.4789 [M-H]$^-$ (765.4789, 0.0)

F_4	15.95	$C_{42}H_{70}O_{12}$	811.4838 [M-H+HCOOH]$^-$ (811.4844, -0.7); 765.4774 [M-H]$^-$ (765.4789, -2.0)
Rk_3	16.24	$C_{36}H_{60}O_8$	665.4264 [M-H+HCOOH]$^-$ (665,4265, -0.2)
Rh_4	16.67	$C_{36}H_{60}O_8$	665.4265 [M-H+HCOOH]$^-$ (665,4265, 0.0)
20(*R*)-Rg_3	18.25	$C_{42}H_{72}O_{13}$	829.4944[M-H+HCOOH]$^-$ (829.4949, -0.6); 783.4891[M-H]$^-$ (783.4895, -0.5);
			621.4373[M-H-(Glc-H_2O)]$^-$ (621.4366, 1.1); 459.3837 [M-H-2(Glc-H_2O)]$^-$ (459.3838, -0.2)
20(*R*)-PPT	18.70	$C_{30}H_{52}O_4$	521.3850 [M-H+HCOOH]$^-$ (521.3842, 1.5)
Rk_1	21.27	$C_{42}H_{70}O_{12}$	811.4848 [M-H+HCOOH]$^-$ (811.4844, 0.5); 765.4783 [M-H]$^-$ (765.4789, -0.8)
Rg_5	21.67	$C_{42}H_{70}O_{12}$	811.4853 [M-H+HCOOH]$^-$ (811.4844, 1.1); 765.4780 [M-H]$^-$ (765.4789, -1.2)

Supplementary Table 5 Contents of the eleven ginsenosides in the 24 h feces of the four group rats (ng/g, *n*=6)

Analyte	Blank	Model	OF	GPs
Re	6093.60 ± 6348.53	121491.70 ± 104632.70*	18243.78 ± 15545.76^{+}	23477.91 ± 15355.90^{+}
Rg_1	218534.90 ± 315766.60	58256.36 ± 60478.57	322962.40 ± 582047.00	67117.65 ± 62744.95
Rf	647.087 ± 470.41	442.13 ± 314.67	783.65 ± 923.21	514.78 ± 226.39
Rb_1	3.71 ± 2.43	2.49 ± 1.84	84.74 ± 156.73	9.19 ± 20.30
20(*S*)-Rg_2	16302.83 ± 10154.32	72229.66 ± 60461.32*	5444.08 ± 5507.11^{+}	13217.92 ± 10363.85^{+}
Rc	45.13 ± 28.75	72.95 ± 35.02*	16.05 ± 8.66^{++}	47.65 ± 13.01^{+}
Rb_2	54.89 ± 39.70	99.62 ± 311.62	1346.50 ± 2637.37	60.93 ± 72.10
Rd	1410.65 ± 668.07	645.57 ± 484.50**	218.59 ± 165.28^{++}	299.44 ± 124.13^{+}
F_2	3063.95 ± 3334.22	1976.36 ± 1090.99	34987.77 ± 55533.93	2392.80 ± 1792.33
20(*S*)-Rg_3	236301.80 ± 64669.82	201318.50 ± 137314.20	376070.00 ± 426707.80	95815.06 ± 55398.23^{+}
CK	714344.80 ± 175067.20	789820.10 ± 190996.60	415378.90 ± 38426.26^{++}	470279.30 ± 173142.50^{++}

*: $p<0.05$, **: $p<0.01$, compared with Blank;

$^{+}$: $p<0.05$, $^{++}$: $p<0.01$, compared with Model.

Supplementary Table 6 Analytical conditions for quantitative determination of the eleven ginsenosides by HPLC-TQ-MS

Analyte	t_R (min)	Channel	MRM	Cone voltage (V)	Collision voltage (eV)
Re	1.92	Ⅰ	m/z 969 → m/z 203	75	55
Rg_1	1.94		m/z 823 → m/z 203	65	40
Digoxin	5.39	Ⅱ	m/z 803 → m/z 283	70	50
Rf	6.08		m/z 823 → m/z 365	70	50
Rb_1	6.73	Ⅲ	m/z 1131 → m/z 365	80	60
20(*S*)-Rg_2	7.07		m/z 807 → m/z 349	75	45
Rc	7.08		m/z 1101 → m/z 335	65	60
Rb_2	7.41		m/z 1101 → m/z 335	65	60
Rd	8.17	Ⅳ	m/z 969 → m/z 203	70	50
F_2	10.55	Ⅴ	m/z 807 → m/z 203	70	45
20(*S*)-Rg_3	11.57		m/z 807 → m/z 365	80	50
CK	14.29		m/z 645 → m/z 203	50	35

Supplementary Table 7 Linearity and sensitivity for the eleven ginsenosides in rat plasma (*n*=3)

Analyte	Linear range (ng/mL)	Equation[a]	R^2	LOD (ng/mL)	LOQ (ng/mL)
Re	0.25-100	y=0.0069x+0.0028	0.9998	0.10	0.25
Rg_1	0.25-100	y=0.0138x+0.0052	0.9992	0.10	0.25
Rf	0.25-100	y=0.0040x+0.0008	0.9994	0.10	0.25
Rb_1	0.25-100	y=0.6417x-0.0209	0.9997	0.10	0.25
20(*S*)-Rg_2	0.25-100	y=0.0021x+0.0011	0.9996	0.10	0.25
Rc	0.25-100	y=0.0347x+0.0042	0.9994	0.10	0.25
Rb_2	0.25-100	y=0.0431x+0.0095	0.9995	0.10	0.25
Rd	0.25-100	y=0.0145x-0.0014	0.9993	0.10	0.25
F_2	0.25-100	y=0.0294x+0.0099	0.9996	0.10	0.25
20(*S*)-Rg_3	0.25-100	y=0.0079x-0.0017	0.9990	0.10	0.25
CK	0.25-100	y=0.0089x+0.0008	0.9991	0.10	0.25

[a]: mean of three standard curves

Supplementary Table 8 Matrix effects, intra-, inter-day precisions and accuracies for the eleven ginsenosides in rat plasma (*n*=3)

Analyte	QC concentration (ng/mL)	Matrix effect		Intra-day		Inter-day	
		Mean (%)	RSD (%)	Precision (RSD %)	Accuracy (RE %)	Precision (RSD %)	Accuracy (RE %)
Re	0.50	87.34	9.01	5.62	-9.56	11.66	+5.55
	2.50	95.97	8.10	5.33	-5.87	5.33	-9.85
	10.00	97.62	4.83	8.75	+5.10	7.56	-0.53
	80.00	85.18	1.46	5.62	-5.67	7.22	-11.54
Rg_1	0.50	88.05	6.10	5.88	-3.61	7.11	-8.85
	2.50	94.22	7.75	10.49	+7.21	4.59	-9.40
	10.00	87.93	4.00	4.46	-14.20	6.74	-11.20
	80.00	98.56	1.64	6.13	+7.48	9.32	+13.42
Rf	0.50	89.49	3.94	12.91	-9.64	12.57	-8.75
	2.50	97.84	2.94	1.59	-12.83	13.72	-0.12
	10.00	94.69	1.49	6.81	-8.10	9.13	-14.09
	80.00	90.78	0.61	1.80	-13.85	9.37	-14.97
Rb_1	0.50	98.90	1.03	2.98	-1.84	5.35	-1.28
	2.50	98.93	3.38	9.63	-1.54	10.89	-4.69
	10.00	96.88	2.97	5.33	-14.28	6.73	-14.62
	80.00	87.45	0.58	1.12	-13.61	5.41	-9.93
20(*S*)-Rg_2	0.50	89.33	6.72	13.22	-10.47	12.55	-9.98

	2.50	87.49	0.97	10.88	-3.46	10.88	-2.75
	10.00	91.04	8.50	5.36	-4.06	3.94	-10.06
	80.00	95.23	5.46	0.57	+0.47	6.90	+3.53
Rc	0.50	91.73	1.48	5.67	-4.22	5.27	-4.33
	2.50	94.69	3.90	13.45	-9.90	5.96	-0.03
	10.00	96.14	5.90	5.96	-9.79	5.91	-3.36
	80.00	98.85	1.02	8.98	-4.81	12.83	-8.04
Rb_2	0.50	96.49	5.47	13.42	-4.07	12.39	-0.05
	2.50	93.56	3.09	14.01	-12.32	9.86	-5.26
	10.00	98.24	2.95	3.48	-14.56	6.37	-12.54
	80.00	85.36	0.57	1.37	-13.17	3.49	-14.65
Rd	0.50	92.05	1.23	7.17	-7.86	4.98	-7.02
	2.50	94.70	8.64	9.48	-14.18	12.00	-14.60
	10.00	91.00	2.08	9.51	-0.61	7.80	+1.28
	80.00	89.35	4.51	1.04	-12.52	1.85	-10.95
F_2	0.50	88.89	3.82	4.43	-8.87	4.82	-12.82
	2.50	98.77	7.73	5.07	-13.72	3.59	-9.92
	10.00	97.80	6.69	0.12	-11.71	7.55	-10.17
	80.00	85.92	0.10	3.59	-8.23	3.59	-8.33
20(*S*)-Rg_3	0.50	85.26	2.06	3.68	-7.91	2.84	-6.53

	2.50	90.94	5.20	11.37	-9.86	12.95	-12.85
	10.00	95.44	1.72	4.07	-9.83	5.98	-8.09
	80.00	87.52	1.94	0.90	-10.39	0.90	-11.19
CK	0.50	86.14	2.88	3.28	+0.37	2.46	-6.08
	2.50	90.79	8.67	3.88	+4.89	4.29	+3.56
	10.00	90.71	9.69	3.89	+7.89	3.39	+0.23
	80.00	96.34	4.39	11.87	-15.85	7.79	-9.99

Supplementary Table 9 Recovery and stability of the eleven ginsenosides in rats plasma (*n*=3)

Analyte	QC concentration (ng/mL)	Recovery		Stability (RSD, %)			
		Mean (%)	RSD (%)	Freeze/thaw stability (-20°C to 25°C)	Autosampler stability (10°C)	Short-term stability (25°C)	Long-term stability (-20°C)
Re	0.50	95.95	8.08	2.53	11.35	14.70	6.93
	2.50	89.43	8.10	9.92	11.30	5.33	8.43
	10.00	94.03	4.83	3.43	6.94	8.75	4.36
	80.00	92.40	6.20	1.74	3.54	5.62	4.24
Rg_1	0.50	89.56	8.97	8.87	6.55	9.73	13.85
	2.50	90.16	8.94	9.68	2.72	5.15	8.94
	10.00	90.31	5.94	8.88	1.92	4.46	8.10
	80.00	99.10	1.64	8.72	7.84	6.13	1.49
Rf	0.50	86.31	13.70	12.92	12.66	14.34	14.03
	2.50	87.11	3.08	4.64	8.97	1.59	8.26
	10.00	91.29	7.55	8.80	5.84	6.81	4.22
	80.00	85.81	0.55	6.45	0.83	1.80	3.77
Rb_1	0.50	93.96	0.92	3.86	5.81	1.87	0.59
	2.50	91.74	3.58	7.93	10.18	0.43	8.52
	10.00	89.16	6.34	3.95	4.03	5.33	4.46
	80.00	86.48	0.85	5.77	1.12	3.71	1.22

20(*S*)-Rg_2	0.50	88.25	5.98	12.53	12.94	14.50	7.44
	2.50	83.55	0.97	3.14	9.26	8.35	4.70
	10.00	89.29	9.55	2.41	1.80	5.36	3.06
	80.00	97.64	3.62	1.02	8.06	0.57	1.22
Rc	0.50	90.28	2.78	6.39	5.57	4.49	5.19
	2.50	99.35	7.31	8.72	5.96	5.01	9.43
	10.00	97.68	8.16	5.61	3.51	5.96	7.20
	80.00	95.62	9.89	9.04	9.99	8.98	6.78
Rb_2	0.50	88.15	5.68	3.70	14.22	5.74	5.03
	2.50	98.25	9.47	2.78	7.10	4.56	7.07
	10.00	88.44	4.57	6.77	0.35	3.48	1.12
	80.00	86.26	0.54	2.61	0.34	1.37	4.33
Rd	0.50	86.82	2.47	2.46	5.73	0.98	2.82
	2.50	92.82	12.73	9.48	10.72	9.48	5.81
	10.00	92.07	2.08	7.68	0.49	9.51	6.03
	80.00	88.11	4.13	1.53	2.26	1.04	1.22
F_2	0.50	85.78	4.58	6.04	5.54	1.61	2.99
	2.50	90.32	6.92	3.91	0.23	9.54	5.58
	10.00	95.69	5.98	9.72	2.96	0.12	5.65
	80.00	91.39	3.90	3.01	2.02	3.59	6.41

20(*S*)-Rg_3	0.50	89.27	1.84	3.14	3.33	1.26	1.38
	2.50	86.61	1.25	9.56	0.62	7.99	3.76
	10.00	91.41	4.71	5.49	5.50	4.07	8.42
	80.00	88.39	1.76	5.26	4.79	0.90	2.43
CK	0.50	93.27	3.37	2.24	2.01	0.68	3.13
	2.50	98.12	5.26	3.49	6.70	3.88	2.57
	10.00	96.35	8.37	10.62	3.39	9.06	6.65
	80.00	85.99	3.94	7.84	3.28	6.42	6.15

Supplementary Figures

Gut microbiota-involved mechanisms in enhancing systemic exposure of ginsenosides by coexisting polysaccharides in ginseng decoction

Shan-Shan Zhou[1, 2, #], Jun Xu[3, #], He Zhu[1], Jie Wu[2], Jin-Di Xu[1], Ru Yan[4], Xiu-Yang Li[1, 2], Huan-Huan Liu[1, 2], Su-Min Duan[2], Zhuo Wang[2], Hu-Biao Chen[3], Hong Shen[2, *], Song-Lin Li[1, 2, *]

Supplementary figure legends

Supplementary Figure 1 Molecular weight distribution (a) and compositional monosaccharide analysis (b: mixed standards; c: sample) of ginseng polysaccharides

Supplementary Figure 2 Chemical structures of ginsenosides and digoxin (the internal standard)

Supplementary Figure 3 OPLS-DA (a: score plot; b: loading plot; c: S-plot) results based on the plasma metabolic profiling of rats in negative (1) and positive (2) ion modes (♦: Blank; +: Model; n=6)

Supplementary Figure 4 PCA (a: score plot) and OPLS-DA (b: loading plot; c: S-plot) results based on the urine metabolic profiling of rats in negative (1) and positive (2) ion modes (♦: Blank; +: Model; n=6)

Supplementary Figure 5 Comparison of potential biomarkers in the plasma (a) and urine (b) between the four group rats (n=6);
*****: $p<0.05$, ******: $p<0.01$, compared with Blank; **+**: $p<0.05$, **++**: $p<0.01$, compared with Model

Supplementary Figure 6 Species accumulation curves (a) and Shannon-Wiener curves (b) of the samples

Supplementary Figure 7 Chemical profiling of 24 h feces in the four group rats after i.g. administration of ginsenoside extracts by UPLC-QTOF-MS analysis

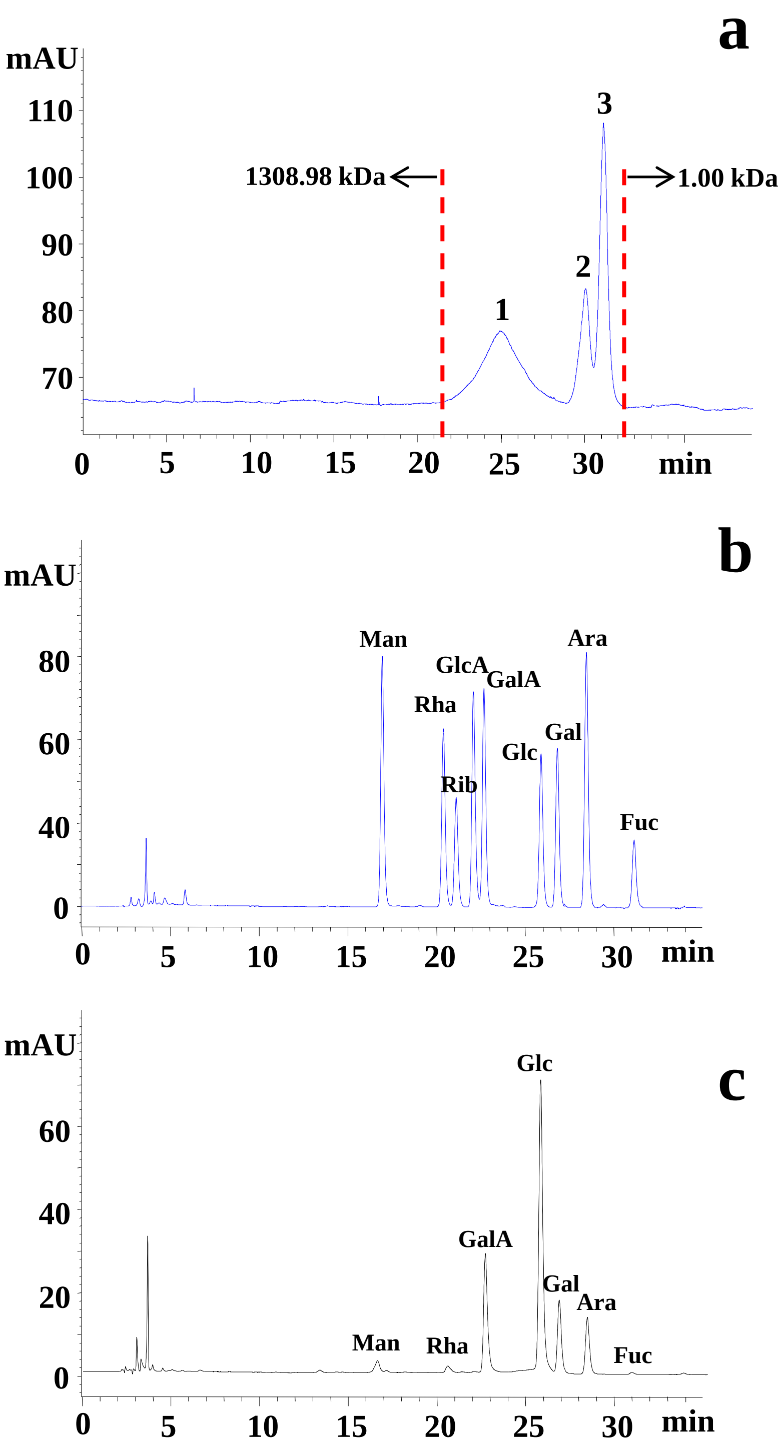

Supplementary Figure 1

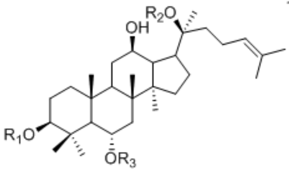

Ginsenoside	R_1	R_2	R_3
Re (**2**)	-H	-Glc	-Glc$^{2-1}$Rha
Rg_1 (**1**)	-H	-Glc	-Glc
Rf (**3**)	-H	-H	-Glc$^{2-1}$Glc
20(*S*)-Rg_2 (**7**)	-H	-H	-Glc$^{2-1}$Rha
20(*S*)-Rh_1 (**9**)	-H	-H	-Glc
F_1 (**17**)	-H	-Glc	-H
S-PPT (**22**)	-H	-H	-H

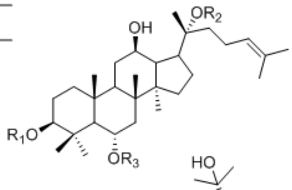

Ginsenoside	R_1	R_2	R_3
R-Noto-R_2 (**6**)	-H	-H	-Glc$^{2-1}$Xyl
20(*R*)-Rg_2 (**11**)	-H	-H	-Glc$^{2-1}$Rha
20(*R*)-Rh_1 (**12**)	-H	-H	-Glc

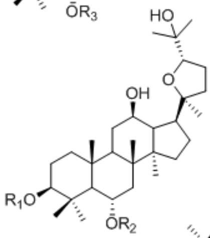

Pseudoginsenoside	R_1	R_2
F_{11} (**4**)	-H	-Glc$^{2-1}$Rha
Rt_5 (**5**)	-H	-Glc

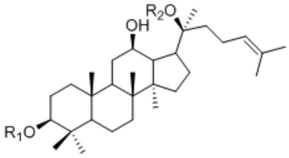

Ginsenoside	R_1	R_2
F_3 (**10**)	-H	-Glc$^{6-1}$Ara(f)
Rb_1 (**8**)	-Glc$^{2-1}$Glc	-Glc$^{6-1}$Glc
Rc (**13**)	-Glc$^{2-1}$Glc	-Glc$^{6-1}$Ara(f)
Rb_2 (**15**)	-Glc$^{2-1}$Glc	-Glc$^{6-1}$Ara(p)
Rb_3 (**16**)	-Glc$^{2-1}$Glc	-Glc$^{6-1}$Xyl
Rd (**18**)	-Glc$^{2-1}$Glc	-Glc
F_2 (**19**)	-Glc	-Glc
Noto-Ft_1 (**20**)	-Glc$^{2-1}$Glc$^{2-1}$Xyl	-H
20(*S*)-Rg_3 (**21**)	-Glc$^{2-1}$Glc	-H
CK (**23**)	-H	-Glc
20(*S*)-Rh_2 (**24**)	-Glc	-H

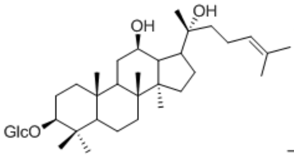

20(*R*)-Rh_2 (**25**)

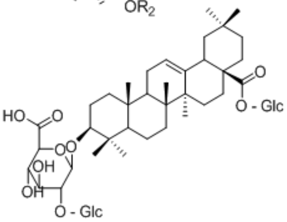

Ginsenoside Ro (**14**)

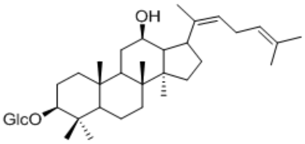

Ginsenoside Rh_3 (**26**)

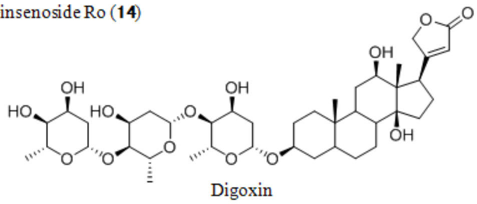

Digoxin

Supplementary Figure 2

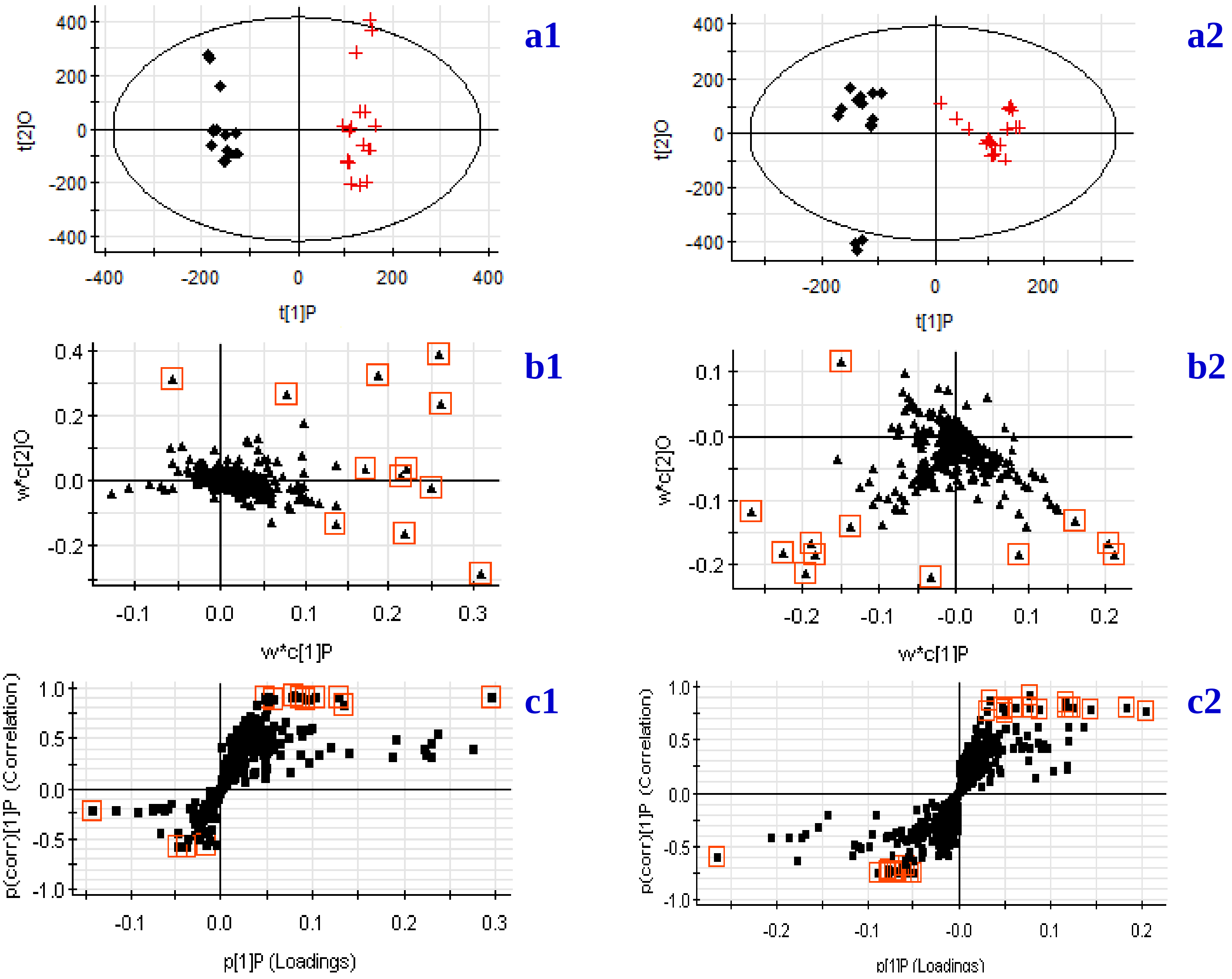

Supplementary Figure 3

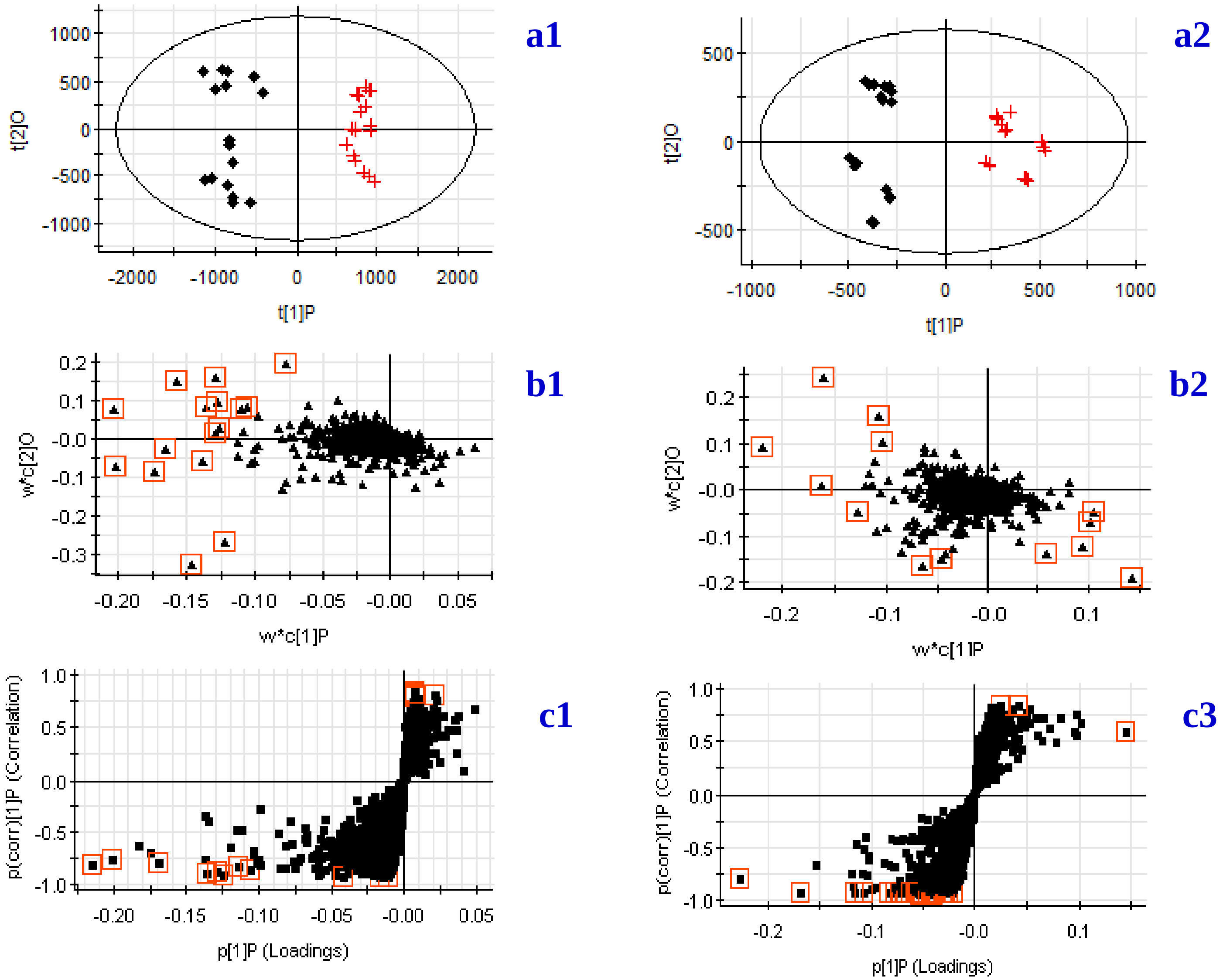

Supplementary Figure 4

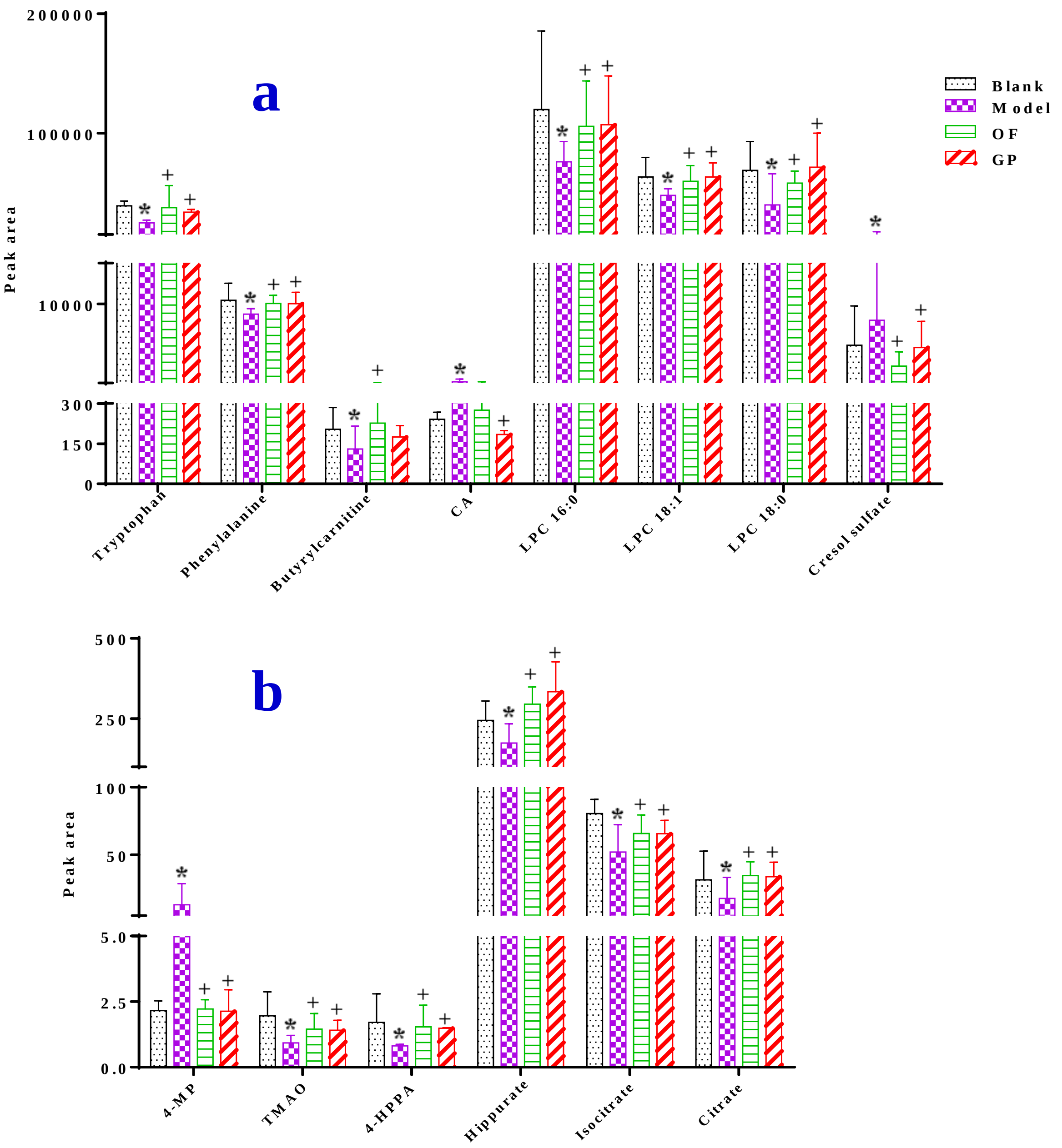

Supplementary Figure 5

a

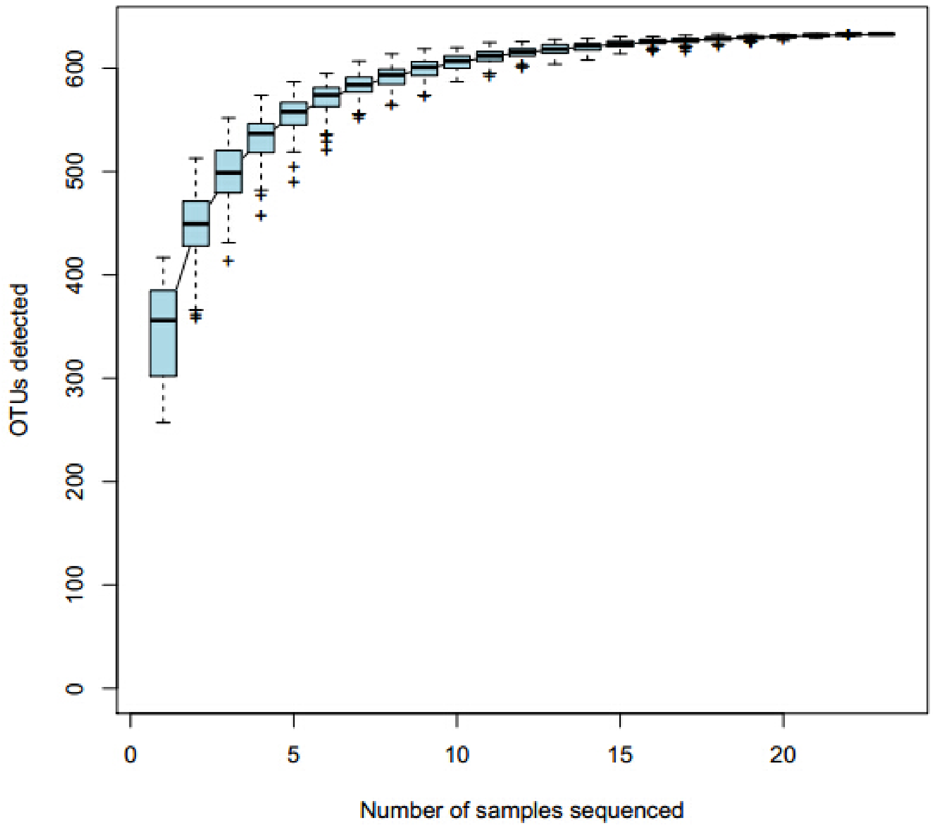

b

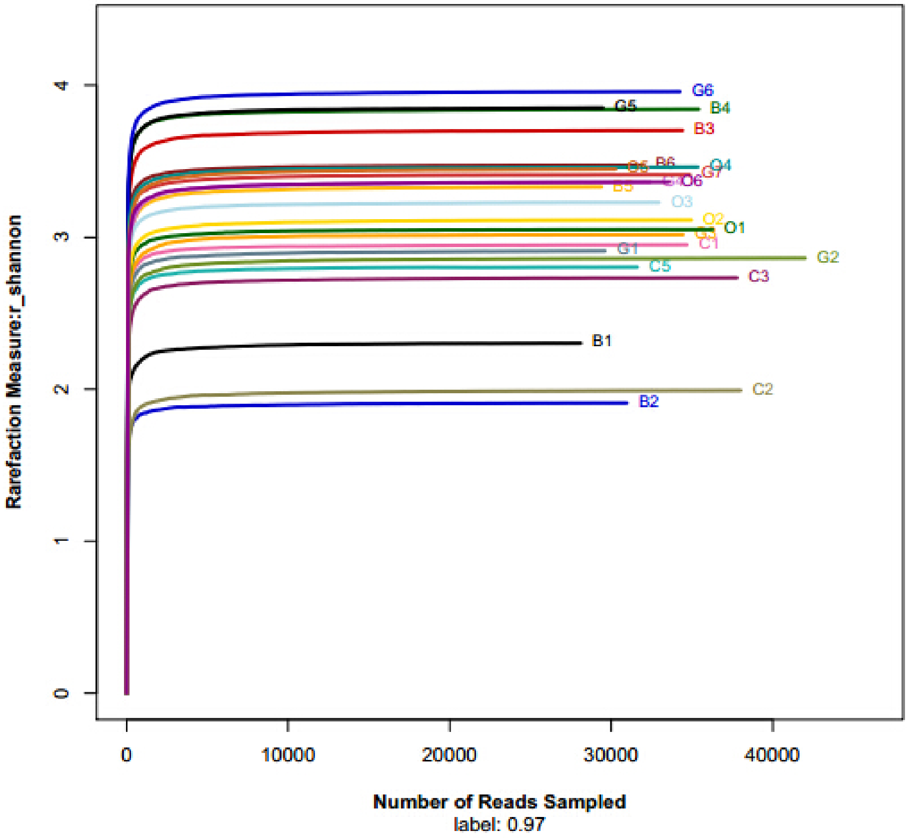

Supplementary Figure 6

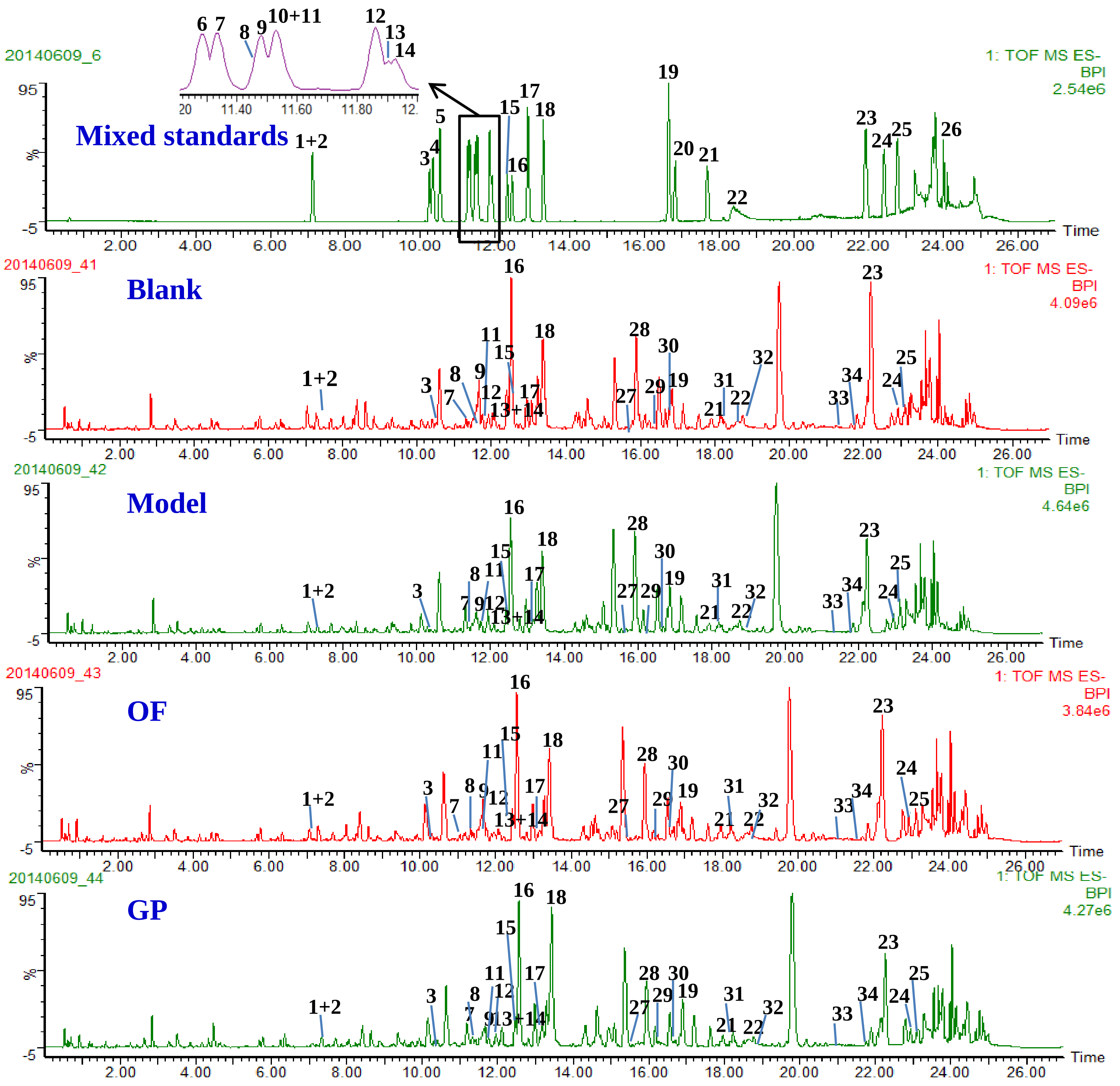

Supplementary Figure 7
